# De novo assembly of *Euphorbia fischeriana *root transcriptome identifies prostratin pathway related genes

**DOI:** 10.1186/1471-2164-12-600

**Published:** 2011-12-13

**Authors:** Roberto A Barrero, Brett Chapman, Yanfang Yang, Paula Moolhuijzen, Gabriel Keeble-Gagnère, Nan Zhang, Qi Tang, Matthew I Bellgard, Deyou Qiu

**Affiliations:** 1 Centre for Comparative Genomics, Murdoch University, WA 6150, Australia; 2 State Key Laboratory of Tree Genetics and Breeding, The Research Institute of Forestry, Chinese Academy of Forestry, Beijing 100091, China; 3 Guangxi Branch Institute, Institute of Medicinal Plant Development, Chinese Academy of Medical Sciences, Nanning 530023, China

## Abstract

**Background:**

*Euphorbia fischeriana *is an important medicinal plant found in Northeast China. The plant roots contain many medicinal compounds including 12-deoxyphorbol-13-acetate, commonly known as prostratin that is a phorbol ester from the tigliane diterpene series. Prostratin is a protein kinase C activator and is effective in the treatment of Human Immunodeficiency Virus (HIV) by acting as a latent HIV activator. Latent HIV is currently the biggest limitation for viral eradication. The aim of this study was to sequence, assemble and annotate the *E. fischeriana *transcriptome to better understand the potential biochemical pathways leading to the synthesis of prostratin and other related diterpene compounds.

**Results:**

In this study we conducted a high throughput RNA-seq approach to sequence the root transcriptome of *E. fischeriana*. We assembled 18,180 transcripts, of these the majority encoded protein-coding genes and only 17 transcripts corresponded to known RNA genes. Interestingly, we identified 5,956 protein-coding transcripts with high similarity (> = 75%) to *Ricinus communis*, a close relative to *E. fischeriana*. We also evaluated the conservation of *E. fischeriana *genes against EST datasets from the Euphorbeacea family, which included *R. communis*, *Hevea brasiliensis *and *Euphorbia esula*. We identified a core set of 1,145 gene clusters conserved in all four species and 1,487 *E. fischeriana *paralogous genes. Furthermore, we screened *E. fischeriana *transcripts against an in-house reference database for genes implicated in the biosynthesis of upstream precursors to prostratin. This identified 24 and 9 candidate transcripts involved in the terpenoid and diterpenoid biosyntehsis pathways, respectively. The majority of the candidate genes in these pathways presented relatively low expression levels except for 1-hydroxy-2-methyl-2-(E)-butenyl 4-diphosphate synthase (HDS) and isopentenyl diphosphate/dimethylallyl diphosphate synthase (IDS), which are required for multiple downstream pathways including synthesis of casbene, a proposed precursor to prostratin.

**Conclusion:**

The resources generated in this study provide new insights into the upstream pathways to the synthesis of prostratin and will likely facilitate functional studies aiming to produce larger quantities of this compound for HIV research and/or treatment of patients.

## Background

*Euphorbia fischeriana *is a perennial herbaceous flowering plant, located in northeast of China. The dried plant roots of *E. fischeriana *and other Euphorbia species are called "Lang-Du", which means *extremely toxic *in Chinese. The roots have often been used in traditional Chinese medicine to treat a wide range of ailments, such as edema, ascites, and cancer [1]. Previous studies of the plant have revealed it mainly contains diterpenoids, triterpenoids and steroids [2]. One particular tigliane diterpene and phorbol ester found in the roots of *E. fischeriana *is 12-deoxyphorbol-13-acetate, commonly known as prostratin.

Prostratin was first discovered during ethno botanical studies carried out on the island of Savai'i, Samoa, where native healers used the bark of *Homalanthus nutans*, a small rain-forest tree from the Euphorbiaceae family, called "mamala" to treat hepatitis infection [3,4]. Prostratin has been shown in previous studies to be a protein kinase C activator [5] and effective in the treatment of HIV infected patients, by acting as a latent HIV1 activator, enabling anti-viral drugs to eliminate a large proportion of viral reservoirs in the body [6,7]. Latent HIV is one of the biggest obstacles HIV researchers face in regards to the treatment of patients [8]. Prostratin has also been found to be an anti-tumour promoter, unlike other diterpene compounds that are known to promote tumorigenesis [9,10].

Very little is known about the biosynthetic pathway of prostratin, although a lot is known about its biochemical structure. The main pathways which are likely to play a role in the synthesis of prostratin and which were chosen for this study were the Terpenoid Backbone Biosynthesis (TBB) pathway (KEGG map00900) and the Diterpenoid Biosynthesis (DB) pathway (KEGG map00904). The TBB pathway is an important pathway for the synthesis of terpenoid or isoprene (5-carbon) compounds that are the building blocks for many important complex compounds synthesised further down the pathway (down stream) in other important biosynthetic pathways. The TBB pathway consists of two separate parallel biosynthetic pathways, one corresponding to the 2-C-methyl-D-erythritol 4-phosphate (MEP) pathway that takes place in the plastids, and another comprising the mevalonic acid (MVA) pathway that occurs in the cytosol. Both pathways lead to the synthesis of terpenoid building blocks: isopentenyl diphosphate (IPP) and dimethylallyl diphosphate (DMAPP). These terpenoid compounds are then used to generate compounds geranyl diphosphate (GPP), farsenyl diphosphate (FPP) and geranylgeranyl diphosphate (GGPP), which are all precursor compounds to pathways further down stream, such as the DB pathway. The DB pathway is an important pathway, which begins with GGPP and leads to the synthesis of many important diterpenoids (20-carbon) compounds. The synthesis of one particular diterpenoid compound that is investigated in this study is casbene, which has a very similar skeleton structure to prostratin. It is highly likely that casbene is a precursor to prostratin [11].

Similar properties to prostratin have been reported for 12-deoxyphorbol 13-phenylacetate (DPP), which has been derived from *Euphorbia resinifera *and very close relative of *E. fischeriana *[12]. DPP has very similar structure to prostratin, with the addition of an ester group at C13, concluding that many more phorbol esters may have anti-HIV properties and would be worth investigating in future studies [13,14].

Cross talk and/or interaction between distinct metabolic pathways are important to determine the potential metabolite profile of a cell under specific conditions. Distinct pathways may share common intermediate compounds for their downstream processing. An associated pathway to the DB pathway is the Kaurenol and the Zeatin Biosynthesis (ZB) pathway (KEGG map00908). Kaurenol is derived from GGPP and serves as a precursor for the synthesis of various gibberellin compounds that act as plant hormones modulating growth and development [15]. The intermediate DMAPP compound of the TBB pathway is utilized to initiate the ZB pathway that leads to the synthesis of Zeatin, a member of the cytokinin family, a class of plant hormones involved in various processes of plant growth and development (KEGG map00908).

In this study we present the results of next generation sequencing, de novo assembly and annotation of the *E. fischeriana *root transcriptome. Based on literature review and KEGG pathway information we identified candidate genes involved in the synthesis of upstream precursors to prostratin and estimated the expression levels of these enzymes.

## Results and Discussion

### Sequencing and de novo transcriptome assembly

Next generation sequencing technologies have significantly facilitated a wide range of genomics applications including high throughput sequencing of non-model plant transcriptomes. To obtain *E. fischeriana *transcriptome expression profiles in roots, and identify candidate genes upstream of prostratin synthesis, where traces of prostratin has been previously reported [1], Illumina technology was used to sequence an *E. fischeriana *library of transcripts expressed in roots generating more than 17.5 million pair-end short reads encoding 1.3 billion bases (Table 1). We initially evaluated the base quality of the sequenced reads (Additional file 1) and trimmed poor quality bases as well as removed poor quality reads (Additional file 2). After trimming we retained 17.1 million high quality pair-end reads and an additional 209,321 single-end reads. The average length of short reads after trimming decreased from 75 bp to 68 bp. To aid in the process of de novo assembly and scaffolding we also sequenced 1,884 high quality ESTs encoding an additional 1.3 million bases (Table 1).

**Table 1:** Summary of *Euphorbia fischeriana *transcriptome assembly

Assembly statistics	
Total number of mate-pair reads (before trimming)	17,502,188
Total number of read base pairs (bp)	1,312,664,100
Average read length (before trimming; bp)	75
Total number of read mate-pairs (after trimming)	17,073,322
Total number of read singletons (after trimming)	209,321
Average read length (after trimming; bp)	68
Total number of ESTs	1,884
Total number of EST base pairs (bp)	1,275,624
Average EST length	677 bp
Total number of transcripts assembled (pre-isoform filtering)	31,454
Total number of transcripts assembled (post-isoform filtering)	18,180
Average length of all transcripts (bp)	1,122
Transcripts with E-value > = 1e^-05 ^against nr	15,191 (83.6%)
Average length of transcripts (bp)	1,066

To determine the best parameters for transcriptome de novo assembly using Oases [16] multiple k-mers were compared (Additional file 3). Our analysis determined that de novo assembly using a k-mer of 25 provided the best compromise between high and low abundant transcripts (see Methods). We also determined that a minimum k-mer coverage threshold of two is suitable for de novo assembly as this removes the majority of sequencing errors (Additional file 4). Thus, a k-mer of 25 and a k-mer coverage cut-off of two were used to assemble the *E. fischeriana *root transcriptome using Oases [16] by combining both high quality Illumina short reads and ESTs in a hybrid assembly approach.

Another feature of Oases is that attempts to cluster assembled transcripts into 'gene clusters' containing two or more putative alternative splicing (AS) isoforms. Our preliminary manual inspection of randomly selected gene clusters revealed that the majority of the predicted AS isoforms corresponded to spurious calls including RNA degradation products, sequence gaps denoted by Ns that were introduced in the scaffolding step and clustering of unrelated sense-antisense transcripts among others (Additional files 5 and 6). These assembly artefacts occur in part due to the extreme variability in coverage depth between genes, isoforms and along each isoform increasing the complexity of the de Brujin graph structure. To remove spurious isoforms from downstream analyses we selected a single transcript from each gene cluster based on various filtering criteria: i) the transcript has the highest Oases confidence score (ratio of nodes in transcript to nodes in gene cluster) that represents the transcripts with the largest number of exons, ii) encodes the longest ORF, iii) corresponds to the longest nucleotide transcript, and iv) in cases where two or more transcripts have the same length then the one with highest sequence coverage was selected. This generated a reference *E. fischeriana *root transcriptome of 18,180 transcripts (Table 1).

### Transcriptome annotation

To determine protein-coding transcripts we screened the *E. fischeriana *root transcriptome against the non-redundant (nr) NCBI peptide database using BLASTx with a cut-off E-value of 1e^-05^. This resulted in 15,191 transcripts (83.6%) annotated as similar to known proteins or matching known conserved hypothetical proteins (Table 2). We also found 819 (4.5%) transcripts harbouring an ORF > = 80 amino acids that represent putative *E. fischeriana*-specific hypothetical protein-coding genes. The remaining 2,171 (11.9%) unannotated transcripts encoded putative short ORFs and may correspond to non-coding RNAs (ncRNAs). To test this notion we subjected these transcripts to tRNAScan-SE [17] and RNAmmer [18] scan. This resulted in the finding of 14 tRNA genes including two pseudogenes encoded in seven transcripts (Table 2 and Additional file 7) and the identification of duplicated copies of the 8S, 18S and 28S rRNAs (Table 2 and Additional file 8). Unannotated transcripts not matching tRNAs and rRNAs may correspond to putative novel ncRNAs or sequencing artefacts, overall we found 2,158 unannotated transcripts (Table 2).

**Table 2:** Statistics of functional annotation of transcripts

Annotated Proteins	# transcripts	% Total transcripts
**a) Protein-coding genes**		
Similar to known proteins	8,834	48.57%
Conserved hypothetical proteins	6,356	34.95%
Hypothetical proteins (ORF > = 80aa)	819	4.50%
Subtotal	16,009	88.06%
**b) Non-coding RNA genes**		
Putative long ncRNAs	2,158	11.87%
tRNA genes	5 (12)^a,b^	0.06%
Pseudo tRNA genes	2	0.01%
rRNA genes	6	0.03%
Subtotal	2,171 (20)^a^	11.94%
Total number of assembled transcripts	18,180 (18,187)^a^	100%

^a^Total number of tRNAs including four tRNAs identified in a transcriptome assembly using a shorter k-mer size of 17 and a minimum transcript length of 100 bp.

^b^transcripts EFI_002280 and EFI_003197 encodes three and two tRNA genes, respectively.

**Figure 1:**
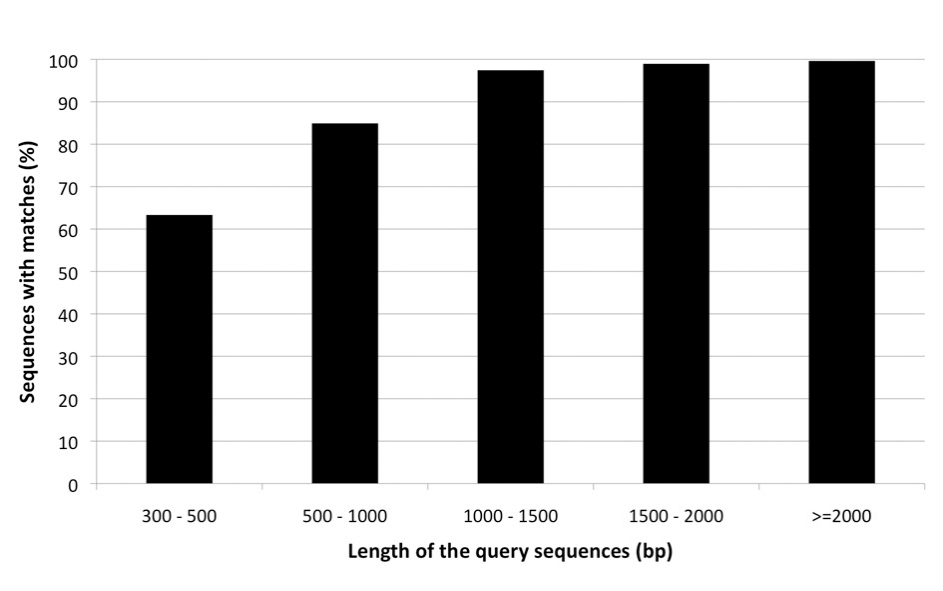
**The effect of query sequence length on the distribution of significant matches against NCBI non-redundant (nr) peptide database**. The number of transcripts with matches (cut-off E-value of 1e^-05^) in NCBI peptide database (nr) is greatest with the longer assembled sequences.

**Figure 2:**
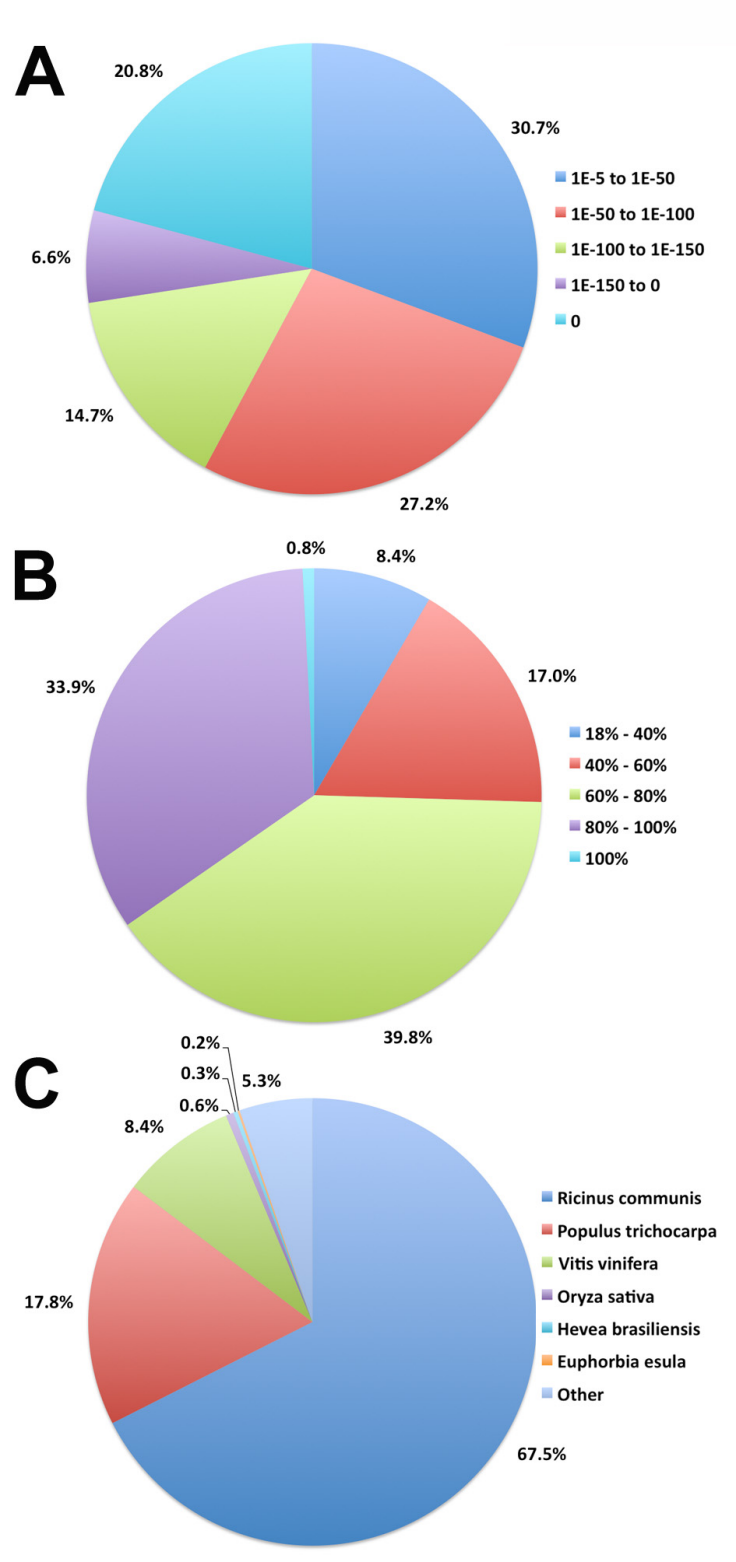
**Statistics of homology search of transcripts against nr peptide database**. A) E-value distribution of the top BLASTx hits with a cut-off E-value of 1e^-05^. B) Similarity distribution of the top BLASTx hits with a cut-off E-value of 1e^-05^. C) Species distribution of the top BLASTx hits is shown as a percentage of the total homologous sequences with an E-value greater than or equal to 1e^-05^.

Figure 1 indicates that the proportion of sequences with matches in the nr database is greater among longer assembled transcripts. Specifically 99.6% of sequences in the > = 2,000 bp range matched to the peptide database, whereas this decreased to 84.7% and 63% for sequences in the 500-1,000 bp and 300-500 bp range, respectively. The E-value distribution of the top hits in the nr database showed that 27% of the mapped sequences have strong similarity (smaller than 1e^-150^), whereas 73% of the homolog sequences ranged from 1e^-5 ^to 1e^-150 ^(Figure 2A). The similarity distribution has a comparable pattern with 35% of the sequences having a similarity higher than 80%, while 65% of the hits have a similarity ranging from 18% to 80% (Figure 2B).

The species distribution showed that the majority of top matches were to *Ricinus communis *(68%), *Populus trichocarpa *(18%) and *Vitis vinifera *(8%) (Figure 2C). The top matches to *R. communis *were further evaluated and identified that 5,956 transcripts were highly similar (> = 75%) to the *E. fischeriana *transcriptome. The BLASTx species distribution showed a bias towards *R. communis *owing to the over representation of this species within the database compared to other species such as *Euphorbia esula*, a closer relative of *E. fischeriana*.

We then evaluated the relative expression levels of *E. fischeriana *transcripts as described in methods and categorized these into three expression ranges 1-125, 125-250 and more than 250 mean coverage expression level. The majority of the transcripts fall into the first category (14,365; 96%), while only 339 (2%) and 261 (2%) were assigned to the 125-250 and 250 plus categories, respectively.

### Functional and pathway annotations

**Figure 3:**
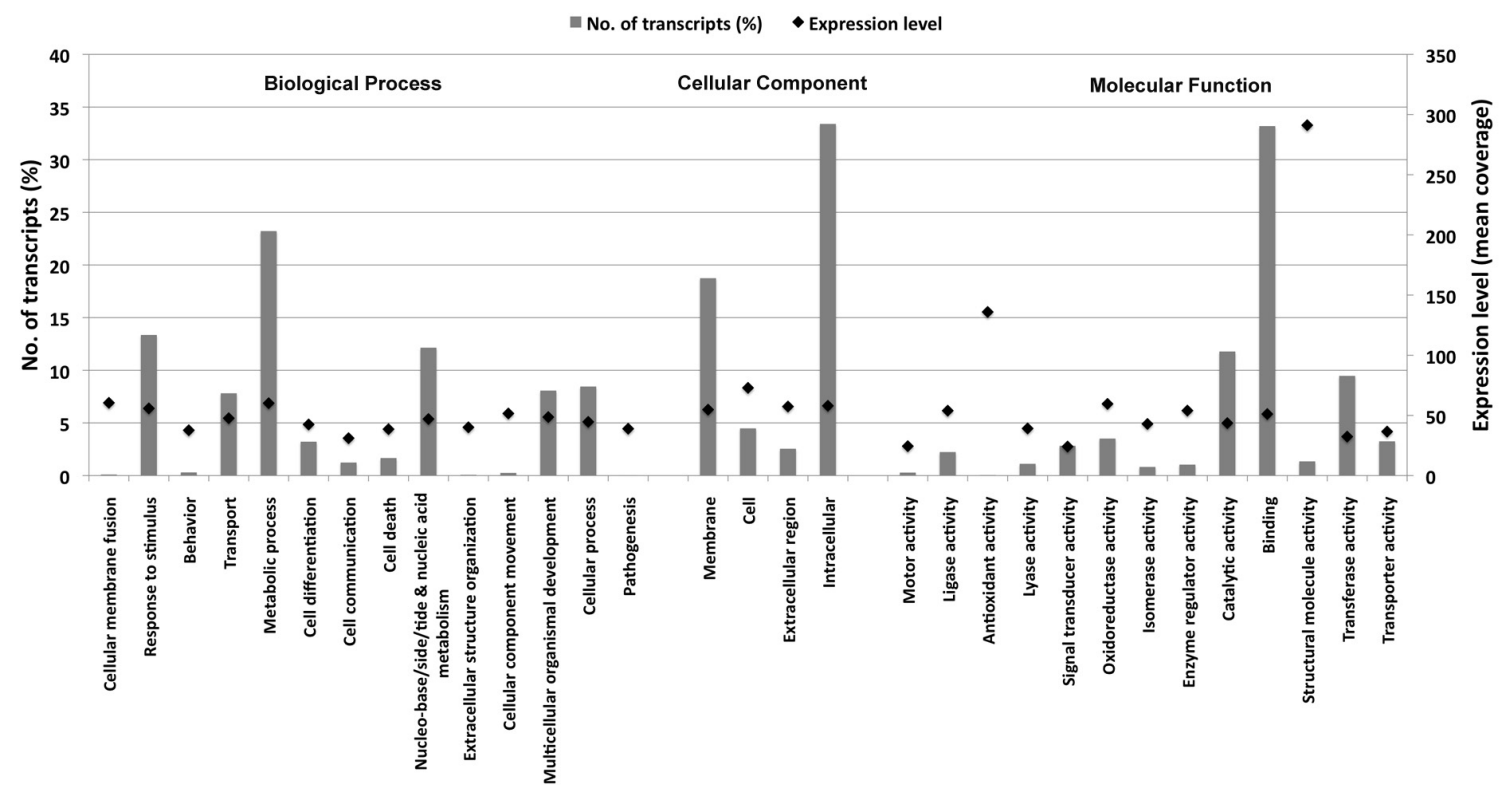
**Frequencies and mean expression levels of transcripts matching GO terms**. The percentage of transcripts matching GO terms is show for each category as grey bars and the normalized mean expression levels of transcripts matching each of these GO terms are shown as black diamonds.

To assign functional information to transcripts Gene Ontology codes were annotated using Annot8r [19]. This yielded 7,841 annotated transcripts (43.1%) covering a broad range of GO categories (Figure 3). The most abundant Biological Process GO codes represented by the largest percent of transcripts were 'Metabolic process' (23.2%) and 'Response to stimulus' (13.4%), indicating that a large range of metabolic activities occur in *E. fischeriana *root. Interestingly, the largest category within metabolic processes corresponds to phosphorylation that encompasses 795 transcripts (4.4%) with the majority of these involved in protein phosphorylation. In roots reversible protein phosphorylation has been implicated in modulating delivery and response to auxin signals [20] that is essential for plant growth and development.

Among the 2,428 transcripts assigned to the response to stimulus category, 370 and 257 were assigned to 'defence response' and 'response to cadmium ion', respectively. Interestingly, 191 of the transcripts assigned to defence response were associated with defence against bacteria, which correlates with the known anti-microbial properties of root extracts from Euphorbia species, such as *Euphorbia fusiformis *[21].

Under Molecular Function GO the two most abundant categories were 'binding' and 'catalytic activity' accounting for 33.2% and 11.8% of the transcripts, respectively (Figure 3). The 6,034 transcripts assigned to the binding category were classified into various categories including protein binding (2,552; 14%), nucleotide binding (1,934; 10.6%), ATP binding (1,708; 9.4%), metal ion binding (1,562; 8.6%), DNA binding (868; 4.8%) and RNA binding (596; 3.3%). The large fraction of transcripts associated with protein, nucleotide and/or metabolite binding suggest the presence of an intricate interactome network in *E. fischeriana *roots.

Next we evaluated the expression levels for each GO code by averaging the coverage of all transcripts matching to each GO code (see Methods). We found that the GO Molecular Function codes 'Structural molecular activity' and 'Antioxidant activity' showed the highest overall expression levels with an average mean coverage of 291 and 136, respectively (Figure 3). Interestingly the majority of the GO Biological Process and Cellular Component codes presented an overall expression level close to 50 (Figure 3).

**Figure 4:**
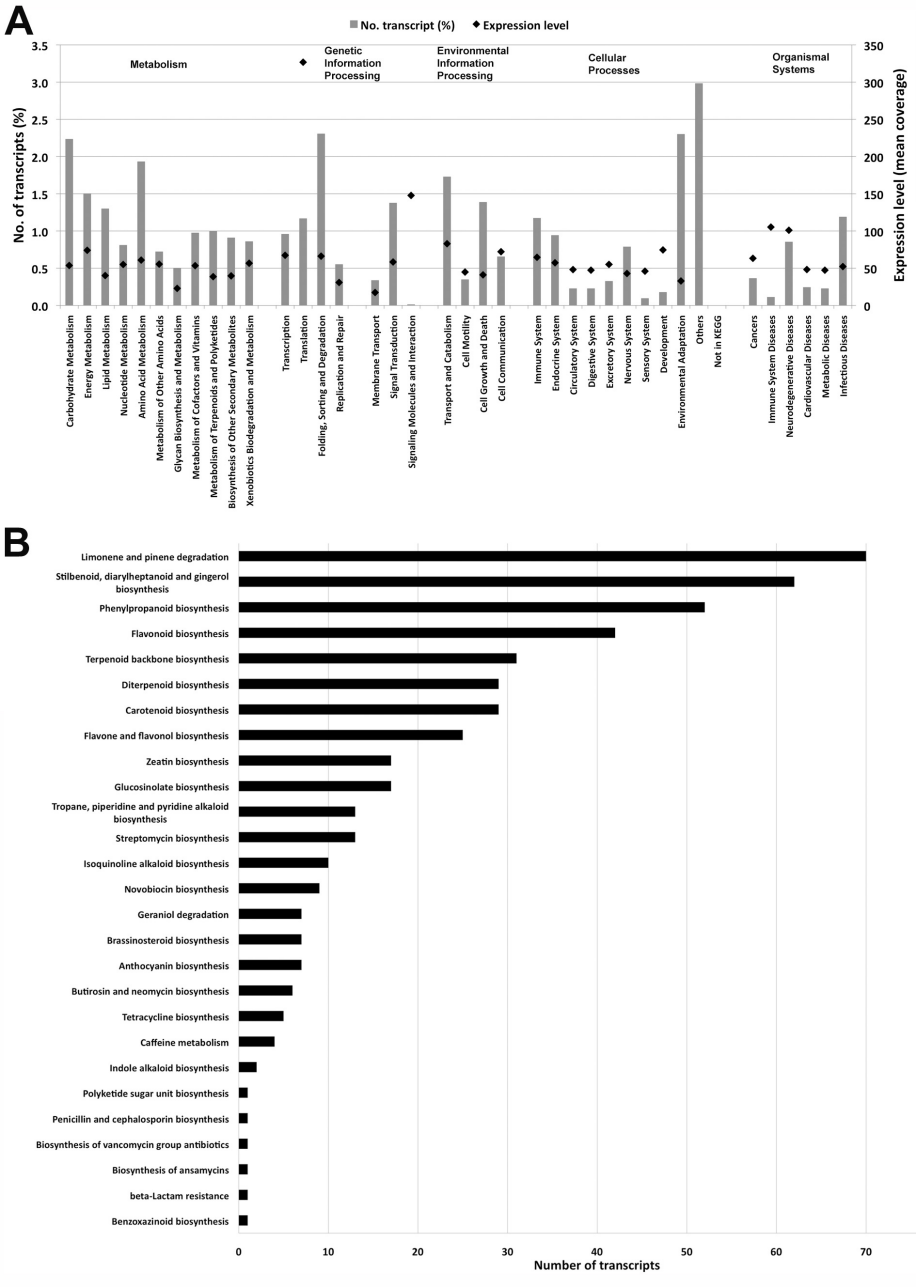
**Frequencies and mean expression levels of transcripts matching KEGG pathways**. The percentage of transcripts matching to KEGG pathways within each high level category are shown as grey bars, while the normalized mean expression levels of transcripts matching each KEGG pathways are indicated as black diamonds. B) Number of transcripts for each secondary metabolite pathways is shown.

The *E. fischeriana *root transcriptome was further annotated by mapping the transcripts onto pathways in Kyoto Encyclopedia of Genes and Genomes (KEGG). A total of 3,189 transcripts (17.5%) were assigned to 293 KEGG pathways, of these 3,103 transcripts were also assigned enzyme commission (EC) numbers. The percentage of assigned transcripts and their overall mean coverage expression level for each of the top 37 KEGG pathways are shown in Figure 4A. The top KEGG metabolic pathways included carbohydrate metabolism, energy and lipid metabolism, amino acid metabolism and the biosynthesis of secondary metabolites. In the secondary metabolism, 270 transcripts were classified into 27 subcategories, and most of them were mapped to terpenoid backbone and diterpenoid biosynthesis, carotenoid biosynthesis, flavone and flavonol biosynthesis, zeatin and glucosinolate biosynthesis (Figure 4B). These results indicated the active metabolic processes in *E. fischeriana *root, but also imply that a variety of metabolites are synthesized in the root. Interestingly as anticipated the terpenoid and diterpenoid biosynthesis pathways encompasses the largest number of transcripts related to secondary metabolites, which may relate with the capacity of *E. fischeriana *root to produce prostratin and related diterpenoid compounds.

We next determined the overall expression levels for each KEGG pathway as described above for GO codes. The pathways with the highest expression levels were 'Translation' and 'Signaling molecules and interaction' with mean coverage values of 327 and 148, respectively (Figure 4A). The majority of all other pathways displayed similar expression levels with an overall mean coverage close to 50.

### Comparison of *E. fischeriana *transcriptome with related species

**Figure 5:**
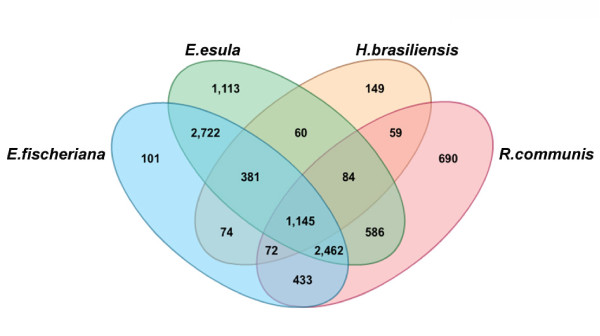
**Euphorbiaceae comparative transcriptome analysis**. The BLAST program tBLASTx was used in conjunction with OrthoMCL using a threshold E-value of 1e^-20 ^to identify orthologous genes between *E. fischeriana *and related species. Sequence datasets from related species were made non-redundant using CD-HIT-EST [33]. The number of orthologous or putative species-unique gene clusters is shown for all comparisons.

To evaluate the conservation of the *E. fischeriana *genes in related species we compared transcripts for each locus against NCBI Expressed Sequence Tags (ESTs) of *Hevea brasiliensis*, *E. esula *and *R. communis*. ESTs were used, as these are the most comprehensive available resource for comparative analyses. Non-redundant sequence datasets for *E. fischeriana *(18, 179 sequences), *E. esula *(32,532 sequences), *H. brasiliensis *(5,457 sequences), and *R. communis *(19,893 sequences) were generated and clustered to identify orthologous gene clusters as described in methods. A total of 10,131 gene clusters were identified among *E. fischeriana*, *E. esula*, *H. brasiliensis *and *R. communis*. Figure 5 shows the overlapping orthologous genes between all evaluated species. As expected we found that *E. fischeriana *shares the largest number of orthologous genes with *E. esula *(6,710; 90.8%) as compared to *R. communis *(4,112; 55.6%) and *H. brasiliensis *(1,672; 22.6%), but in terms of proportion of shared orthologous sequences as compared to the total number of available genes for each species, *H. brasiliensis *shared the largest fraction of their genes (30.6%) as compared to the equality similar fractions of 20.6% and 20.7% for *E. esula *and *R. communis*, respectively. We also found 1,145 genes that are shared by all species representing a potential core orthologous gene set for Euphorbiaceae species. To characterize further this core gene set, we evaluated their assigned Gene Ontology (GO) codes. In terms of GO biological processes we found 43% and 24% transcripts annotated under 'Metabolic Processes' and 'Response to stimulus', respectively. Other important categories were 'Nucleobase, nucleoside, nucleotide and nucleic acid metabolic process' (18%), 'Cellular process' (14.3%), 'Multicellular organismal development' (13.97%) and 'Transport' (13.2%). We also identified 1,487 paralog genes within *E. fischeriana *transcriptome. Our results provide a preliminary overview of core genes shared between Euphorbiaceae species based on currently available resources. We anticipate that this dataset will be expanded and refined further as more significant transcriptome sequencing efforts are conducted in other Euphorbiaceae species.

### Candidate genes upstream of prostratin biosynthesis pathway

**Figure 6:**
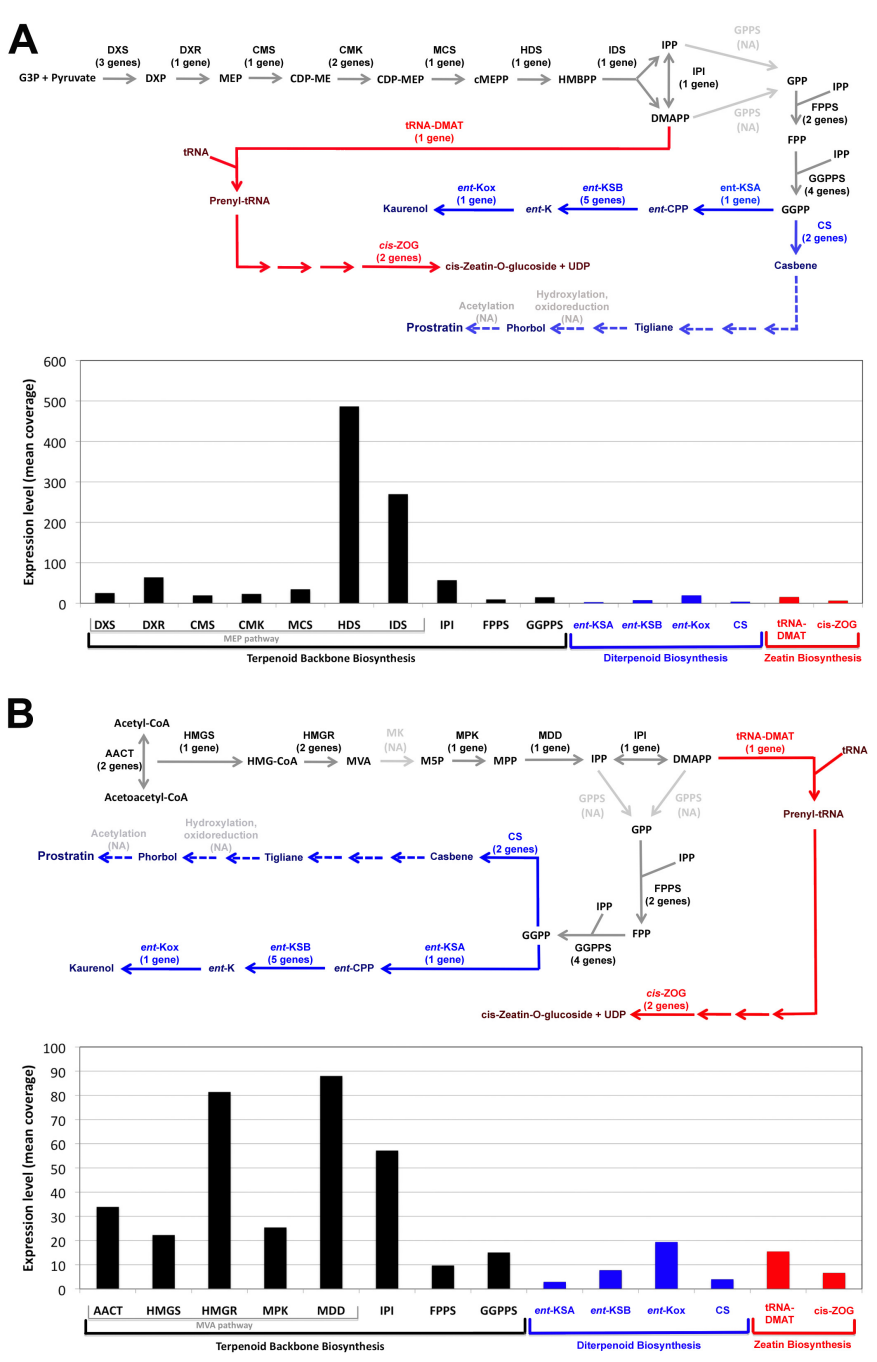
**Mean expression levels within the Terpenoid Backbone, Diterpenoid and Zeatin Biosynthesis pathways**. A) Normalized mean expression levels for enzymes within the Terpenoid Backbone Biosynthesis (TBB) pathways, namely, plastidic 2-*C*-methyl-*D*-erythritol 4-phosphate (MEP) pathway, Diterpenoid Biosynthesis (DB) pathway and Zeatin Biosynthesis (ZB) pathway are provided. B) Normalized mean expression levels for enzymes within the TBB pathways, namely, cytosolic mevalonic acid (MVA) pathway, DB and ZB pathways. Number of *E. fischeriana *transcripts, from distinct gene clusters, matching each enzyme are shown between brackets in panels A and B. Abbreviations: AACT, acetoacetyl-coenzyme A (CoA) thiloase; CMS, 2-*C*-methyl-erythritol 4-phosphate cytidyl transferase; DXR, 1-deoxy-*D*-xylulose 5-phosphate reductoisomerase; DXS, 1-deoxy-*D*-xylulose-5-phosphate synthase; FPPS, farnesyl diphosphate synthase; GGPPS, geranylgeranyl diphosphate synthase; GPPS, geranyl diphosphate synthase; NA, Not Available; HMGR, 3-hydroxy-3-methylglutaryl coenzyme A (HMG-CoA) reductase; IPI, isopentenyl diphosphate isomerase; MK, mevalonate kinase; MPK, mevalonate-5-phosphate kinase; CMK, 4-(cytidine 5'-diphospho)-2-*C*-methyl-*D*-erythritol kinase; MDD, mevalonate diphosphate decarboxylase; IDS, isopentenyl diphosphate/dimethylallyl diphosphate synthase; MCS, 2-*C*-methyl-*D*-erythritol 2,4-cyclodiphosphate synthase; HDS, 1-hydroxy-2-methyl-2-(*E*)-butenyl 4-diphosphate synthase; HMGS, HMG-CoA synthase; HMG-CoA, 3S-hydroxy-3-methylglutaryl coenzyme A; DXP, 1-deoxy-*D*-xylulose 5-phosphate; MVA, 3R-Mevalonic acid; M5P, Mevalonate-5-phosphate; MPP, Mevalonate diphosphate; MEP, 2-*C*-methyl-*D*-erythritol 4-phosphate; CDP-ME, 4-(cytidine 5'-diphospho)-2*C*-methyl-*D*-erythritol; CDP-MEP, 4-(cytidine 5'-diphospho)-2*C*-methyl-*D*-erythritol 2-phosphate; cMEPP, 2*C*-methyl-*D*-erythritol 2,4-cyclodiphosphate; DMAPP, Dimethylallyl diphosphate; HMBPP, 1-hydroxy-2-methyl-2-(*E*)-butenyl 4-diphosphate; IPP, isopentenyl diphosphate; G3P, Glyceraldehyde 3-phosphate; GPP, geranyl diphosphate; GGPP, geranylgeranyl diphosphate; FPP, farnesyl diphosphate; GGPPS, geranylgeranyl diphosphate; *ent*-KSA, *ent*-Kaurene synthase A; *ent*-KSB, *ent*-Kaurene synthase B; *ent*-Kox, *ent*-Kaurene oxidase; CS, Casbene synthase; tRNA-DMAT, tRNA Dimethylallyltransferase; *cis*-ZOG, *cis-*Zeatin O-beta-D-glucosyltransferase; *ent*-CPP, *ent*-Copalyl diphosphate; *ent*-K, *ent*-Kaurene; UDP, Uridine 5'-diphosphate.

Prostratin is a phorbol ester from the tigliane diterpene series. Recently casbene a product of the DB pathway (KEGG map00904) has been shown to be structurally similar to prostratin [11]. The DB pathway requires geranylgeranyl diphosphate (GGPP) as a precursor for casbene synthesis. To characterise possible candidate genes upstream of prostratin synthesis we screened the *E. fischeriana *transcriptome for enzymes in the TBB (KEGG map00900) and DB pathways. We found 24 and 9 transcripts encoding the candidate genes in the TBB and DB pathways, respectively (Figure 6).

Transcripts matching to genes encoding enzymes involved in the TBB, DB and ZB pathways were found through BLASTx nr searches using an E-value threshold of 1e^-05 ^(Figure 6A). We found two transcripts, encoding for geranylgeranyl diphosphate synthase (GGPPS) and 3-hydroxy-3-methylglutaryl coenzyme A reductase (HMGR) contained transcripts on the opposite strands encoding different genes, not classified as TBB genes. These transcripts were further evaluated to determine if these corresponded to Naturally occurring Antisense Transcripts (NATs), but these were not NATs (Additional file 6). Furthermore, this highlighted another artefact produced using the Oases tool when unrelated transcripts may be clustered into the same cluster.

The DB pathway is of particular interest, as one of the downstream products is the phorbol ester prostratin. All diterpenes begin synthesis from GGPP, a 20-carbon isoprenoid diphosphate, derived primarily from the MEP pathway (Figure 6A). GGPP is also the precursor to many other compounds in plants, such as chlorophylls, prenylated proteins and gibberellins [22,23]. On entry to the DB pathway, GGPP is converted to a variety of diterpenes by diterpene synthases. Only a hand full of diterpene synthases have been identified thus far, including casbene and neocembrene synthases from *R. communis *and other well known Euphorbiaceae species [24,25].

Casbene is considered the most likely precursor to prostratin [11], although other diterpenes with similar structures could possibly go through a further series of structural changes with the same end product, so other possible paths within the DB pathway were considered such as the initial structural changes of GGPP, to *ent*-copalyl diphosphate and then *ent*-kaurene. A number of transcripts coding for these enzymes involved in this path were found with BLASTx nr searches and their expression levels were evaluated based on the mean read coverage of these transcripts as described previously.

The expression levels of enzymes in the TBB and DB pathways were compared to those of enzymes in the ZB pathway. We found that the TBB pathway showed an overall higher expression level as compared to other downstream pathways. Interestingly 1-hydroxy-2-methyl-2-(E)-butenyl 4-diphosphate synthase (HDS) and isopentenyl diphosphate/dimethylallyl diphosphate synthase (IDS) from the MEP pathway presented the highest expression levels (Figure 6A). Comparison of the MEP and MVA pathways within the TBB pathway revealed that genes in the MVA pathway had lower overall expression levels, although enzymes such as 3-hydroxy-3-methylglutaryl coenzyme A reductase (HMGR) and mevalonate diphosphate decarboxylase (MDD) had higher relative expression levels. These findings suggest that overall within the TBB pathway, there is a preference for the synthesis of larger amounts of dimethylallyl diphosphate (DMAPP) and isopentenyl diphosphate (IPP) that is required to drive various downstream pathways including diterpene synthesis (Figure 6A).

To validate the observed RNA-seq expression trends we selected 16 transcripts encoding 8 enzyme types and designed primers for real time RT-PCR (Table 3). As shown in Additional file 9, we determined a very strong correlation of expression for GGPPS, DXS, AACT, HMGR, MDD, IDS and HDS. The only exception was casbene (CS) that showed a relatively high expression level in real time RT-PCR in contrast to the very low expression detected in our RNA-seq experiment (Figure 6). These findings indicate that although a good correlation was observed for the majority of the enzymes tested and global trends can be interpreted, it is required to conduct independent validations to accurately measure the expression level of enzymes of interest.

**Table 3:** Real time PCR primers used for expression validations of selected enzymes.

Accession	Enzyme	5'-Forward Primer Sequence-3'	5'-Reverse Primer Sequence-3'
EFI_002990	AACT-1	ACTATGCTTGCAGCCCAAAG	ATTTCCCATGCCAACATCAT
EFI_012483	AACT-2	ACAATGCTTGCTGCACAGAC	TCTCCACAAACTCCCATTCC
EFI_015339	CS-1	GGAGAGCTATTTTTGGGCAGT	CGACTTGAGCAAATGAGTCGT
EFI_018002	CS-2	GCAATTGATCCATCAGCAAG	AAGCAAAACAACTCTGGCAAT
EFI_007143	DXS-1	CGCACTAAATTTTGGGTTGC	CAAATCCCTTGGAATTGGTG
EFI_010574	DXS-2	GCTGCAAAAAGCATCACAAA	GGAGCTGGCATTGCTTTTAC
EFI_003135	DXS-3	TTTGCAACAAGTGGCATCTC	ATAGCCAAAGCCTCCACAAA
EFI_010535	GGPPS-1	CAAAAAGCTTCGCAATTCCT	GATTTTTGCGGGTTCTCTGA
EFI_010585	GGPPS-2	ACTTGCAGCCGTTTGTTTCT	ATCAGCAACGAGGGAAAATG
EFI_008533	GGPPS-3	ATTGTTAGCGGGTGCTGAAG	CAGCTCTTCCGCCATTTCTA
EFI_016937	GGPPS	TCAATTCGCTGTTCTGCTTC	CCCTTAGAAAGGGCGGAGTA
EFI_001905	IDS/HDR	CCACAGACGACTCTGCTTCA	GGTGTGCTCATTCCCATTTT
EFI_000087	HDS	GTTTGGGCGATACAATCAGG	ATCCACCTCTTCACCCTCCT
EFI_0 00846	HMGR-1	CTCCACCGCAAAACCTCTTA	AACGACATGGAGAGGAGTGG
EFI_011656	HMGR-2	CAGTGCTGTGAAATGCCTGT	AGCTCTTGTCATGCCATCCT
EFI_001179	MDD	GAGACATGGGTGAGGATGGT	CCTCCCCATTAAGCCACATA
EFI_014705	Actin-1	GGGAACGAGTCCCTGGTAGT	CTGCGTTGGTGGTCTTACCT
EFI_006680	Actin-2	AAATATGGCCGACAGTGAGG	ATACCTCGCTTGGACTGAGC
EFI_000139	α-tubulin	GGCAACTTTTCCATCCTGAG	TCCAAGACCAGAACCAGTCC
EFI_014153	β-tubulin-1	AAGCAGGTCAATGTGGGAAC	CATTGTCCCTGGTTCAAGGT
EFI_014578	β-tubulin-2	GCTTGCAAGGTTTCCAGGTA	TTTTCCACAAGCTGATGCAC

There were no transcripts with sequence similarity to geranyl diphosphate synthase (GPPS) identified in this study (Figure 6A and 6B). This may be due to the low expression level of GPPS and the inefficient assembly of poorly expressed genes. GPPS enzyme is critical for the synthesis of geranyl diphosphate (GPP), which is essential for synthesis of farnesyl diphosphate (FPP) via farnesyl diphosphate synthase (FPPS). FPPS was detected in the transcriptome and its expression was found to be relatively low (Figure 6A and 6B). Additionally, mevalonate kinase (MK) transcripts were not found in the transcriptome even though downstream enzyme transcripts were present (Figure 6B). This may be due to similar reasons for the absence of GPPS. As both GPPS and MK enzymes are present in the related *H. brasiliensis *species and stored in public databases under the accession numbers AB294710 and AB294693, respectively. These findings emphasis the need to increase the amount of sequencing data and/or the evaluation of lower k-mer values to de novo assemble low expressed genes. Overall we identified 26 enzymes involved in terpenoid and diterpenoid biosynthesis, including two casbene synthases that are a valuable resource for further biochemical and functional studies leading to increase the production of prostratin.

## Conclusion

The de novo assembly of the *E. fischeriana *root transcriptome identified 18,180 transcripts, of these 15,191 encoded genes with sequence similarity in other species and 1,487 represent paralogous genes. This study identified 26 transcripts encoding enzymes involved in various pathways upstream of the casbene biosynthesis pathway, which is a proposed precursor for prostratin. Furthermore we revealed the high expression of HDS and IDS enzymes in the TBB pathway. Critically we found a significant higher expression level of the *ent*-Kaurene oxidase (*ent*-Kox) and tRNA Dimethylallyltransferase (tRNA-DMAT) enzymes driving the synthesis of kaurenol and cis-zeatin-O-glucoside + UDP, which compete for available GGPP and DMAPP, respectively. DMAPP is essential for the synthesis of GGPP further downstream, while GGPP is directly essential for the synthesis of casbene. The resources generated in this study will likely facilitate further functional studies aiming to increase the production of prostratin, DPP and other phorbol esters of interest for the advancement of HIV research and/or treatment of patients.

## Methods

### Plant material, RNA isolation and deep sequencing

Live plants of *E. fischeriana *were collected in June 2008 from Jiagedaqi of Hei longjiang province of China. The plants were then grown in the green house of Chinese Academy of Forestry, Beijing. The root of *E. fischeriana *was washed with tap water and cut into small pieces. The root materials were immediately frozen in liquid nitrogen and were stored at -80°C until further processing.

Total RNA was isolated according to the method described by Chang et al. [26]. After the RNA pellets were dried, RNA was dissolved in 500 μL of RNase free water. Total RNA purity was checked with Agilent 2100 Nano drop machine. The RNA was stored in a -80°C freezer before being sent to the Beijing Genome Institute (BGI) at Shenzhen with dry ice for mRNA purification and cDNA construction.

The library for transcriptome sequencing was constructed with Illumina's kit following manufacturer's protocol. The mRNA was purified from 10 μg of total RNA using oligo (dT) magnetic beads. After purification, the mRNA was fragmented into small pieces using divalent cations under elevated temperatures. The RNA fragments were used for first strand cDNA synthesis with random primers. Second strand cDNA synthesis was done by using DNA polymerase I and RNaseH. The cDNA fragments then went through an end repair process and were ligated to adapters. The products were purified and enriched with PCR before sequencing on the Illumina GAII sequencing platform. Image deconvolution and quality value calculations were performed using the Illumina GA pipeline 1.3.

### RNA isolation and EST sequencing

Frozen root samples stored at -80°C were sent to the Beijing Genome Institute (BGI) at Beijing on dry ice. Total RNA was isolated as described above. The RNA was stored in a -80°C freezer until further processing.

Approximately 1 μg of total RNA was used for preparing a cDNA library using the Creator Smart cDNA Library construction kit (Clontech, Mountain View, CA, USA) following manufacture's instructions. The resulting second cDNA strand products were then run on an agarose gel and those with a size between 1-3 kbp were excised and purified using the QIAquick PCR Purification kit (QIAGEN, Shanghai, China) according to the manufacturer's protocol. The products were transformed into DH10B competent cells. Library was checked with a titer of 2 × 10^5 ^pfu/mL and a capacity of 1.2 × 10^6 ^clones. A total of 2,099 ESTs were sequenced using capillary sequencing. Vector sequences were removed and 1,884 good EST sequences with an average length of 677 bp and a minimum length of 101 bp were submitted to dbEST at GenBank. The assigned accession numbers are the following: [GenBank:JK523124] to [GenBank:JK525007].

### Transcriptome assembly

We evaluated several assemblers for the de novo assembly of the *E. fischeriana *root transcriptome, including Oases [16], Velvet [27], QSRA [28], Euler-SR [29], Edena [30] and SOAPdenovo [31]. Preliminary assembled contigs by each tool were blasted against NCBI non-redundant protein database. We found that Oases (Velvet version 1.0.12 and Oases version 0.1.15) was the tool with the largest number of database hits (data not shown) and was selected for downstream analyses.

The reads were first trimmed using the adaptive trimming function of a trimming perl script implemented by Nik Joshi at The Bioinformatics Core, UC Davis Genome Centre http://wiki.bioinformatics.ucdavis.edu/index.php/Trim.pl. Additional files 1 and 2 show the results of quality assessment using FastQC prior and after trimming of poor bases and/or removal of poor reads [32], respectively. To assess the best parameters to use for this assembly, multiple assemblies from k-mer 17 to 47 were compared based on N50, the number of transcripts and the number of gene clusters (Additional file 3). A k-mer of 25 was determined to be the best k-mer, with the highest N50, highest number of transcripts and the highest number of gene clusters. A minimum transcript size of 100 bp was also compared to 300 bp for all assemblies in the comparison. The appropriate k-mer coverage cut off was determined using an R package plotrix (Additional file 4). All assemblies used a minimum k-mer coverage of 2× and a pair-end insert size of 200 bp was used and the assembly was assisted using 1,884 *E. fischeriana *ESTs. All other parameters were used on default settings. The final assembly used for annotation used a minimum transcript length of 300 bp.

In many instances Oases predicted spurious isoforms and to increase confidence in the assembled sequences a single transcript from each gene cluster was selected based on the following criteria: i) the transcript has the highest Oases confidence score (ratio of nodes in transcript to nodes in a gene cluster) that represents the transcript with the largest number of exons, ii) encodes the longest ORF, iii) corresponds to the longest nucleotide transcript, and iv) in cases where two or more transcripts have the same length then the one with highest sequence coverage is selected. This generated a dataset of 18,180 transcripts, of these 9,883 transcripts were submitted to GenBank/DDBJ/EMBL following their submission guidelines under the project ID 66759 and Locus Tag EFI. The remaining 8,297 sequences contained gaps denoted by 'Ns' that were introduced during the scaffolding step using pair-end short reads with an expected insert size of 200 bp. A fasta file of all 18,180 transcripts is presented in the Additional file 10.

### Transcriptome annotation

#### Protein-coding genes

Annotation by peptide sequence was done by searching transcripts against the NCBI non-redundant (nr) peptide database which includes all non-redundant GenBank CDS translations, RefSeq Proteins, PDB, SwissProt, PIR and PRF, excluding environmental samples from WGS projects. The search was conducted using BLASTx with an E-value cut-off of 1e^-05 ^and matching to the top hits.

#### RNA genes

The assembled transcripts were scanned for the presence of tRNA and rRNA sequences using the programs tRNAscan-SE [17] and RNAmmer [18], respectively. The tRNA transcripts were predicted from the original assembly using a k-mer of 25 and a minimum transcript size of 300 bp. To identify additional tRNAs we conducted a new assembly using a shorter k-mer of 17 and a minimum transcript length of 100 bp. Newly assembled transcripts were then screened for tRNAs as described above.

#### Gene Ontology (GO) and KEGG pathways

The GO and KEGG annotations were carried out using the annotation program Annot8r [19], which assigned GO and KEGG pathway terms to the transcripts. The program requires a prepared MySQL database and the transcripts in a fasta file. The user progresses through a series of menus, selecting fasta file name, database name, E-value cut off and number of top hits. The assembled transcripts were annotated using an E-value cut off of 1e^-20 ^and the top 5 hits were used for the annotation of each sequence.

### Related species comparison analysis

The EST datasets of closely related species, namely *E. esula*, *H. brasiliensis *and *R. communis *were downloaded from the NCBI EST database. Non-redundant datasets were then generated using CD-HIT-EST as previously described [33]. This yielded non-redundant sequence datasets for *E. fischeriana *(18,179 sequences), *E. esula *(32,532 sequences), *H. brasiliensis *(5,457 sequences), and *R. communis *(19,893 sequences). Sequence similarity comparisons and clustering were performed using tBLASTx in conjunction with OrthoMCL [34] using a defined E-value cut off of 1e^-20^.

### Expression analysis and prostratin candidate genes

To determine the relative expression levels of *E. fischeriana *transcripts high quality trimmed short reads were mapped onto these transcripts using the Burrows-Wheeler Aligner (BWA) [35] and coverage for each nucleotide was determined using SAMtools [36]. The mean coverage for each transcript was then calculated by averaging the coverage for each nucleotide within the transcript. The expression levels of transcripts were then categorized into various expression ranges.

An in-house database of prostratin pathway related candidate genes was created by interrogating the literature and KEGG pathways for genes matching to the TBB, DB and the comparative downstream pathway, the ZB pathway. We then screened *E. fischeriana *transcripts against this in-house database using BLASTx to identify significant matches to enzymes in the TBB (including MEP and MVA pathways), DB and ZB pathways. The ZB pathway, which has little relevance to the synthesis of prostratin and other diterpenes, was chosen for use as a comparison to the DB pathway, to compare other possible competing downstream pathways. The mean coverage values for all transcripts were plotted to determine the changes in expression levels through the pathways.

### RNA isolation, reverse transcription and Real-time PCR

*E. fischeriana *total RNA was isolated from the roots using Column Plant RNAout kit (TIANDZ, Beijing, China), according to the manufacturer's protocol. RNA was treated with DNase I (Takara, Dalian, China) to remove residual genomic DNA. The concentration of the isolated RNA and the 260/280-absorbance ratio was measured in triplicates with Nanodrop ND-8000 (Thermo, USA). The quality of RNA samples was confirmed by electrophoresis on a 1.2% agarose. Total RNA was reverse-transcribed to cDNA using PrimeScript RT reagent Kit (Perfect Real Time) (Takara, Dalian, China) in a total volume of 10 μl, according to the manufacturer's instruction. About 600 ng of total RNA, 2 μl 5 × PrimeScript buffer (for Real Time), 0.5 μl PrimeScript RT Enzyme Mix I, 0.5 μl Oligo dT Primer (50 μM) and 2 μl Random 6 mers (100 μM) were mixed. The reaction was carried out at 37°C for 15 min and 85°C for 5 s.

Several enzymes from the Terpenoid Biosynthesis pathway (Figure 6) that showed various levels of expression were selected for validation using real time PCR. Forward (Fwd) and reverse (Rev) primers were designed using Primer3 as described previously [37]. Table 3 shows the primers for the selected enzymes and controls. The Real-time PCR assays were conducted in an optional 96-well plate with ABI7500 system (ABI, USA) and a commercial SRBR-Green master mix kit (Takara, Dalian, China), according to the manufacturer's protocol. The reactions were performed with 1 μl cDNA in 20 μl reaction mix containing 10 μl 2 × SYBR Premix Ex Taq and 1.0 μl primers. The conditions were as follows: initial holding at 95°C for 3 min, followed by a two-step program of 95°C for 15 s and 58°C for 33 s for 40 cycles. Each sample was analyzed in three technical replicates and mean Ct values were calculated. Reverse transcriptase negative controls and "no template controls" (without cDNA in PCR) were included.

## Abbreviations

HIV: Human Immunodeficiency Virus; DPP: 12-deoxyphorbol 13-phenylacetate; EST: Expressed Sequence Tag; GO: Gene Ontology; KEGG: Kyoto Encyclopedia of Genes and Genomes; ORF: Open Reading Frame; BWA: Burrows-Wheeler Aligner; AS: alternative splicing; AACT: acetoacetyl-coenzyme A (CoA) thiloase; CMS: 2-*C*-methyl-erythritol 4-phosphate cytidyl transferase; DXR: 1-deoxy-*D*-xylulose 5-phosphate reductoisomerase; DXS: 1-deoxy-*D*-xylulose-5-phosphate synthase; FPPS: farnesyl diphosphate synthase; GGPPS: geranylgeranyl diphosphate synthase; GPPS: geranyl diphosphate synthase; HMGR: 3-hydroxy-3-methylglutaryl coenzyme A (HMG-CoA) reductase; IPI: isopentenyl diphosphate isomerase; MK: mevalonate kinase; MPK: mevalonate-5-phosphate kinase; CMK: 4-(cytidine 5'-diphospho)-2-*C*-methyl-*D*-erythritol kinase; MDD: mevalonate diphosphate decarboxylase; IDS: isopentenyl diphosphate/dimethylallyl diphosphate synthase; MCS: 2-*C*-methyl-*D*-erythritol 2,4-cyclodiphosphate synthase; HDS: 1-hydroxy-2-methyl-2-(*E*)-butenyl 4-diphosphate synthase; HMGS: HMG-CoA synthase; HMG-CoA: 3S-hydroxy-3-methylglutaryl coenzyme A; DXP: 1-deoxy-*D*-xylulose 5-phosphate; MVA: 3R-Mevalonic acid; M5P: Mevalonate-5-phosphate; MPP: Mevalonate diphosphate; MEP: 2-*C*-methyl-*D*-erythritol 4-phosphate; CDP-ME: 4-(cytidine 5'-diphospho)-2*C*-methyl-*D*-erythritol; CDP-MEP: 4-(cytidine 5'-diphospho)-2*C*-methyl-*D*-erythritol 2-phosphate; cMEPP: 2*C*-methyl-*D*-erythritol 2,4-cyclodiphosphate; DMAPP: Dimethylallyl diphosphate; HMBPP: 1-hydroxy-2-methyl-2-(*E*)-butenyl 4-diphosphate; IPP: isopentenyl diphosphate; G3P: Glyceraldehyde 3-phosphate; GPP: geranyl diphosphate; GGPP: geranylgeranyl diphosphate; FPP: farnesyl diphosphate; GGPPS: geranylgeranyl diphosphate; *ent*-KSA: *ent*-Kaurene synthase A; *ent*-KSB: *ent*-Kaurene synthase B; *ent*-Kox: *ent*-Kaurene oxidase; CS: casbene synthase; tRNA-DMAT: tRNA Dimethylallyltransferase; *cis*-ZOG: *cis-*zeatin O-beta-D-glucosyltransferase; *ent*-CPP: *ent*-Copalyl diphosphate; *ent*-K: *ent*-Kaurene; UDP: Uridine 5'-diphosphate; TBB: Terpenoid Backbone Biosynthesis; DB: Diterpenoid Biosynthesis; ZB: Zeatin Biosynthesis.

## Authors' contributions

DQ, RB and BC contributed with the conception and design of the research. YY and NZ performed real-time RT-PCR experiment. QT and YY collected plant material and isolated RNA samples for deep sequencing. BC carried out all bioinformatics analysis. PM and GK assisted the bioinformatics analysis. RB and BC conducted data analysis and wrote the manuscript. DQ and MB provided critical review of the manuscript and interpretations. All authors read and approved the final manuscript.

## Supplementary Material

Additional file 1 **Assessment of reads using FastQC before trimming**. A) Quality of reads per base. The central red line is the median base quality, the yellow box represents the inter-quartile range (25-75%), the upper and lower whiskers represent the 10% and 90% points and the blue line represents the mean base quality. B) Distribution of mean quality scores over all sequenced reads. C) The GC content distribution over all sequenced reads as compared against the theoretical GC distribution. The blip in GC content above the theoretical GC distribution is most likely due to the utilized primers at the 5' end of the reads used during RNA-seq library preparation and sequencing. Click here for file

Additional file 2 **Assessment of reads using FastQC after trimming**. A) Quality of reads per base after adaptive window trimming using a quality average threshold of 20 and a minimum length threshold of 20, The central red line is the median value, the yellow box represents the inter-quartile range (25-75%), the upper and lower whiskers represent the 10% and 90% points and the blue line represents the mean base quality B) The mean sequence quality scores over all reads. C) The GC content distribution over all sequenced reads as compared against the theoretical GC distribution. Click here for file

Additional file 3 **Comparison of Oases assemblies using various k-mers**. A) Oases assemblies using k-mers ranging from 17 to 47 with minimum transcript size of 100 bp. B) Oases assemblies with minimum transcript size of 300 bp. C) Zoomed in view of Oases assemblies with minimum transcript size of 300 bp over k-mers 19 to 37. The maximum N50, largest number of gene clusters and largest number of transcripts can be obtained using a k-mer of 25. Thus, this k-mer and a length threshold of > = 300 bp were selected as parameters to assemble the reference *E. fischeriana *root transcriptome. Click here for file

Additional file 4 **Evaluation of k-mer coverage for Velvet assembly**. The frequency of k-mers (number of appearances or coverage) was determined using untrimmed Illumina reads and ESTs. The results show a large peak of k-mers with coverage of one, which mostly correspond to sequencing errors. Thus, a k-mer coverage threshold of 2 was utilized in the de novo transcriptome assembly. Click here for file

Additional file 5 **Example of an Oases 'gene cluster'**. A) Multiple sequence alignment of transcripts into the same 'gene cluster'. Note that transcript 2 (T2) is a 5'end truncation version of T6 and that T4 has a significant sequence variation. B) Blast homology screening revealed that T1, T3 and T5 are mitochondria encoded acetyl-CoA acetyltransferase transcripts. Click here for file

Additional file 6 **Clustering of GGPS with unrelated hypothetical proteins**. A) Blast results of transcripts clustered into EFI_010585 isoform cluster using Oases. Note that only transcript 4 has similarity to GGPS and this is encoded in the reverse strand. B) Multiple sequence alignment of the reverse complemented GGPS transcript and a sequence representing the hypothetical transcripts. Click here for file

Additional file 7 **Predicted tRNA genes in the *E. fischeriana *root transcriptome**. To identify **t**RNA genes the reference assembled root transcriptome was screened using tRNAscan-SE as previously described [17]. Additionally, we identified another four tRNA genes highlighted with asterisks in a transcriptome assembly conducted using a k-mer size of 17 and a length threshold of > = 100 bp. Their fasta sequences are appended at the bottom. Click here for file

Additional file 8 **Predicted rRNA genes the *E. fischeriana *root transcriptome**. To find rRNAs the reference root transcriptome was screened using RNAmmer as previously described [18]. Click here for file

Additional file 9 **Validation of RNA-seq expression trends using a real time RT-PCR approach**. Reciprocal (1/Ct) real time PCR values were averaged for each enzyme mentioned in Table 3. The multiplied by 10000 and log_2 _transformed. Similarly RNA-seq mean average values were log2 transformed and compared against real time RT-PCR results. A) A significantly strong correlation (R^2 ^= 0.91686) of RNA-seq and real time PCR expression levels were observed for six enzymes. From left to right black boxes correspond to GGPPS, DXS, AACT, HMGR, MDD and IDS enzymes; B) Upon inclusion of the HDS expression data (blue box), the linear correlation of RNA-seq and real time PCR expression levels was still significant (R^2 ^= 0.79392); C) Upon further addition of the CS expression data (red box) the linear correlation dropped significantly (R^2 ^= 0.2173). Abbreviations of enzymes are as shown in Figure 6's legend. Click here for file

Additional file 10 **Fasta sequences of 18,180 transcripts**. Click here for file
