# Supplementary material for: De novo assembly of Euphorbia fischeriana root transcriptome identifies prostratin pathway related genes
Source: BMC Genomics. 2011 Dec 13;12:600. doi: 10.1186/1471-2164-12-600 (PMC3273484; doi:10.1186/1471-2164-12-600)
Supplement: Additional file 5 — Example of an Oases 'gene cluster'. A) Multiple sequence alignment of transcripts into the same 'gene cluster'. Note that transcript 2 (T2) is a 5'end truncation version of T6 and that T4 has a significant sequence variation. B) Blast homology screening revealed that T1, T3 and T5 are mitochondria encoded acetyl-CoA acetyltransferase transcripts. [file 1471-2164-12-600-S5.DOC]

**Additional file 5. Example of an Oases ‘gene cluster’.** A) Multiple sequence alignment of transcripts into the same gene cluster. Note that transcript 2 (T2) is a 5’end truncation version of T6 and that T4 has a significant sequence variation. B) Blast homology screening revealed that T1, T3 and T5 are mitochondria encoded acetyl-CoA acetyltransferase transcripts.

**A) Example of multiple sequence alignment of an Oases ‘gene cluster’**

EFI_002990_T3 -------CTGCAGCCTCCGAAATAAAGCCTAAAGATGTTTGCATAGTTGGTGCTGCCCGT

EFI_002990_T5 -------CTGCAGCCTCCGAAATAAAGCCTAAAGATGTTTGCATAGTTGGTGCTGCCCGT

EFI_002990_T1 -------CTGCAGCCTCCGAAATAAAGCCTAAAGATGTTTGCATAGTTGGTGCTGCCCGT

EFI_002990_T6 -------CTGCAGCCTCCGAAATAAAGCCTAAAGATGTTTGCATAGTTGGTGCTGCCCGT

EFI_002990_T2 ------------------------------------------------------------

EFI_002990_T4 GTTCCCTTAAAAATTTCTCGCATTACTCCTTTGCCTGCCTCTCTCTTCATTTCTCCCCCT

EFI_002990_T3 ACGCCAATGGGCGGATTTC-TTGGTTCACTATCATCTTTAACTGCGACCAAGCTCGGATC

EFI_002990_T5 ACGCCAATGGGCGGATTTC-TTGGTTCACTATCATCTTTAACTGCGACCAAGCTCGGATC

EFI_002990_T1 ACGCCAATGGGCGGATTTC-TTGGTTCACTATCATCTTTAACTGCGACCAAGCTCGGATC

EFI_002990_T6 ACGCCAATGGGCGGATTTC-TTGGTTCACTATCATCTTTAACTGCGACCAAGCTCGGATC

EFI_002990_T2 ------------------------------------------------------------

EFI_002990_T4 -CTCTCTCAAACGAACGCGACTGCGGAACCCTAAACTTCAACTTCGAT--GNNNNNNNNN

EFI_002990_T3 TATAGCCATTGGAGCTGCTCTTAAAAGAGCCAATATCGATCCATCACTGGTCCAAGAAGT

EFI_002990_T5 TATAGCCATTGGAGCTGCTCTTAAAAGAGCCAATATCGATCCATCACTGGTCCAAGAAGT

EFI_002990_T1 TATAGCCATTGGAGCTGCTCTTAAAAGAGCCAATATCGATCCATCACTGGTCCAAGAAGT

EFI_002990_T6 TATAGCCATTGGAGCTGCTCTTAAAAGAGCCAATATCGATCCATCACTGGTCCAAGAAGT

EFI_002990_T2 ---------TAGAGCTGCTCTTAAAAGAGCTAATATCAATCCATCACTAGTCCAAGATGT

EFI_002990_T4 NNNNNNNNNNNNNNNNNNNNNNNNNNNNNCCGCCATCGATGTTCCTCCGACCTATGATGT

* *** ** * * * * ** **

EFI_002990_T3 TTTCTTTGGCAATGTTCTAAGTGCAAATTTAGGACAGGCTCCTGCTAGACAGGCTGC---

EFI_002990_T5 TTTCTTTGGCAATGTTCTAAGTGCAAATTTAGGACAGGCTCCTGCTAGACAGGCTGC---

EFI_002990_T1 TTTCTTTGGCAATGTTCTAAGTGCAAATTTAGGACAGGCTCCTGCTAGACAGGCTGC---

EFI_002990_T6 TTTCTTTGGCAATGTTCTAAGTGCAAATTTAGGACAGGCTCCTGCTAGACAGGCTGC---

EFI_002990_T2 TTTCTTTGGCTATGTTCTAAGTGCAAATTTAGGACAGGCTCCTGCTAGACAGGCTGC---

EFI_002990_T4 GTTCGGAGACACCTTCCTCCTTCCCTGCCTCAATCTT-CTCTTTCTCGTCTCTCTCCCTC

*** * * * ** * * * * *** * ** * * ** *

EFI_002990_T3 -ACTAGGTG----CAGGAATTCCTAATTCAG-TAGTCTGCACCACTGTTAACAAAGTTTG

EFI_002990_T5 -ACTAGGTG----CAGGAATTCCTAATTCAG-TAGTCTGCACCACTGTTAACAAAGTTTG

EFI_002990_T1 -ACTAGGTG----CAGGAATTCCTAATTCAG-TAGTCTGCACCACTGTTAACAAAGTTTG

EFI_002990_T6 -ACTAGGTG----CAGGAATTCCTAATTCAG-TAGTCTGCACCACTGTTAACAAAGTTTG

EFI_002990_T2 -ACTAGGTG----CAGGAATTCCTAATTCAG-TAGTCTGCACCACTGTTAACAAAGTTTG

EFI_002990_T4 AACTAACTGTCTCCGGCGACTCCAGATTTAGACAGACGATGGCATGAAACATCATGTAAT

**** ** * * * *** *** ** ** * ** * * **

EFI_002990_T3 TGCTTCGGG-GATGAAAGCAACTATGCTTGCAGCCCAAAGCATCCAGCTAGGCATCAATG

EFI_002990_T5 TGCTTCGGG-GATGAAAGCAACTATGCTTGCAGCCCAAAGCATCCAGCTAGGCATCAATG

EFI_002990_T1 TGCTTCAGG-GATGAAAGGCG--------GGAACTGGTATTATT--GTTGCTTTGCTTTT

EFI_002990_T6 TGCTTCGGG-GATGAAAGCAACTATGCTTGCAGCCCAAAGCATCCAGCTAGGCATCAATG

EFI_002990_T2 TGCTTCGGG-GATGAAAGCAACTATGCTTGCAGCCCAAAGCATCCAGCTAGGCATCAATG

EFI_002990_T4 TTCTGCAAGAGATCGAAGACGA-GCAACCAGAGATCTTGCCGCCGGTCTCCTTCGTTCTC

* ** * * *** *** * * *

EFI_002990_T3 ATGTTGTTGTTGCTGGGGGCATGGAAAGCATGTCCAATGTTCCTAAATATTTGGCAGAAG

EFI_002990_T5 ATGTTGTTGTTGCTGGGGGCATGGAAAGCATGTCCAATGTTCCTAAATATTTGGCAGAAG

EFI_002990_T1 GTGTAGTTGATGCATAATATAC--------------------------------------

EFI_002990_T6 ATGTTGTTGTTGCTGGGGGCATGGAAAGCATGTCCAATGTTCCTAAATATTTGGCAGAAG

EFI_002990_T2 ATGTTGTTGTTGCTGGGGGCATGGAAAGCATGTCCAATGTTCCTAAATATTTGGCAGAAG

EFI_002990_T4 GCAGAGTTTGCCAAAAAGGGATAACTATGCTGTCCAGAGTTT-TGAGCGTGGTATTGCTG

*** *

EFI_002990_T3 CAAGGAAGGGATCTCGACTTGGACATGATTCCCTAGTAGATGGAATGT-TGAAAGATGGG

EFI_002990_T5 CAAGGAAGGGATCTCGACTTGGACATGATTCCCTAGTAGATGGAATGT-TGAAAGATGGG

EFI_002990_T1 ------------------------------------------------------------

EFI_002990_T6 CAAGGAAGGGATCTCGACTTGGACATGATTCCCTAGTAGATGGAATGT-TGAAAGATGGG

EFI_002990_T2 CAAGGAAGGGATCTCGACTTGGACATGATTCCCTAGTAGATGGAATGT-TGAAAGATGGG

EFI_002990_T4 CTCAGGAAAGTGGTTGCTTTGCATGGGAAATTACACCGGTTGAAGTATCTGGGGGAAGGG

EFI_002990_T3 --------TTATGGGATGTTTACAATGATGTTGGCATGGGAAATTGTG---CTGAAATAT

EFI_002990_T5 --------TTATGGGATGTTTACAATGATGTTGGCATGGGAAATTGTG---CTGAAATAT

EFI_002990_T1 ------------------------------------------------------------

EFI_002990_T6 --------TTATGGGATGTTTACAATGATGTTGGCATGGGAAATTGTG---CTGAAATAT

EFI_002990_T2 --------TTATGGGATGTTTACAATGATGTTGGCATGGGAAATTGTG---CTGAAATAT

EFI_002990_T4 GAAAGCCCTCAACAATTGTTGATAAGGATGAAGGTTTAGGAAAGTTTGACCCTGCAAAAT

EFI_002990_T3 GTGCAGATAATCATTCAATTACTAGGGAGGACCAGGATAACTATGCTATC--CAGAGTTT

EFI_002990_T5 GTGCAGATAATCATTCAATTACTAGGGAGGACCAGGATAACTATGCTATC--CAGAGTTT

EFI_002990_T1 ------------------------------------------------------------

EFI_002990_T6 GTGCAGATAATCATTCAATTACTAGGGAGGACCAGGATAACTATGCTATC--CAGAGTTT

EFI_002990_T2 GTGCAGATAATCATTCAATTACTAGGGAGGACCAGGATAACTATGCTATC--CAGAGTTT

EFI_002990_T4 TGAGGAAGCTCCGGCCAAGTTTTAGAGAAAATGGAGGCACAGTTACTGCTGGCAATGCCT

EFI_002990_T3 TGAGCGTG-GTATTGCTGCTCAGGAAAGTGGTTGCTTTGCATGGGAAATTACACCGGTTG

EFI_002990_T5 TGAGCGTG-GTATTGCTGCTCAGGAAAGTGGTTGCTTTGCATGGGAAATTACACCGGTTG

EFI_002990_T1 ------------------------------------------------------------

EFI_002990_T6 TGAGCGTG-GTATTGCTGCTCAGGAAAGTGGTTGCTTTGCATGGGAAATTACACCGGTTG

EFI_002990_T2 TGAGCGTG-GTATTGCTGCTCAGGAAAGTGGTTGCTTTGCATGGGAAATTACACCGGTTG

EFI_002990_T4 CCAGCATAAGTGATGGTGCTGCTGCTTTGGTTTTAGTGAGTGGAGAGACAGCACTTAAGC

EFI_002990_T3 AAGTATCTGGGGGAAGGGGAAAGCCCTCAACAATTGTTGATAAGGATGAAGGTTTAGGAA

EFI_002990_T5 AAGTATCTGGGGGAAGGGGAAAGCCCTCAACAATTGTTGATAAGGATGAAGGTTTAGGAA

EFI_002990_T1 ------------------------------------------------------------

EFI_002990_T6 AAGTATCTGGGGGAAGGGGAAAGCCCTCAACAATTGTTGATAAGGATGAAGGTTTAGGAA

EFI_002990_T2 AAGTATCTGGGGGAAGGGGAAAGCCCTCAACAATTGTTGATAAGGATGAAGGTTTAGGAA

EFI_002990_T4 TTGGACTGGAAGTGATTGCAAAGATCACTGGATATGCTGATGCTGCTCAGGCACCAGAGC

EFI_002990_T3 AGCACTGGAGGGCACT-GCTCGTGCATTGTTCAGGGGATTAAGAGCAGGCCCGGTGCTCT

EFI_002990_T5 AGCACTGGAGGGCACT-GCTCGTGCATTGTTCAGGGGATTAAGAGCAGGCCCGGTGCTCT

EFI_002990_T1 ------------------------------------------------------------

EFI_002990_T6 AGTTTGACCCTGCAA--AATTGAGGAAGCTCCGGCCAAGTTTTAGAGAAAATGGAGGCAC

EFI_002990_T2 AGTTTGACCCTGCAA--AATTGAGGAAGCTCCGGCCAAGTTTTAGAGAAAATGGAGGCAC

EFI_002990_T4 TGTTTACAACGGCTCCTGCCCTGGCGATACCTAAAGCTTTGTCAAAAG--CTAGCTTGGA

EFI_002990_T3 TGTCAGTTCTGGT--TTGCTTGATCTCATGTAGAAGCTCCACTCTAGAGGTTGTGACAA-

EFI_002990_T5 TGTCAGTTCTGGT--TTGCTTGATCTCATGTAGAAGCTCCACTCTAGAGGTTGTGACAA-

EFI_002990_T1 ------------------------------------------------------------

EFI_002990_T6 AGTTACTGCTGGCAATGCCTCCAGCATAAGTGATGGTGCTGCTGCTTTGGTTTTAGTGAG

EFI_002990_T2 AGTTACTGCTGGCAATGCCTCCAGCATAAGTGATGGTGCTGCTGCTTTGGTTTTAGTGAG

EFI_002990_T4 TGCTTCTGAAGTTGATTATTATGAGATAAATGAAGCCTTTGCTGTTGTAGCTCTGTCCAA

EFI_002990_T3 TGCTGTGAAAGCAT--------------GGAAGCTC---TG--------GTCTCA-A--T

EFI_002990_T5 TGCTGTGAAAGCATATTCTACAGCTTGAGAAAATGT---TGCAAAATCAGTTTCA-AGTT

EFI_002990_T1 ------------------------------------------------------------

EFI_002990_T6 TGGAGAGACAGCACTTAAGCTTGGACTGGAAGTGAT---TGCAAAGATCACTGGATATGC

EFI_002990_T2 TGGAGAGACAGCACTTAAGCTTGGACTGGAAGTGAT---TGCAAAGATCACTGGATATGC

EFI_002990_T4 T-CAGAAACTGCTTGGACTTAATCCAGAAAAAGTGAATGTACATGGTGGAGCTGTTTCCT

EFI_002990_T3 TATCTCCACCTTCTAACTCCTATGGTTCCTCTCATTTTCTCTTTT-------TGTGTAG-

EFI_002990_T5 CAATACGATCTCGAGAAGTCAGTGGGTTATAAGATTTCATCTCTTGAAAATCTGGCTAA-

EFI_002990_T1 ------------------------------------------------------------

EFI_002990_T6 TGATGCTGCTCAGGCACCAGAGCTGTTTACAACGGCTCCTGCCCTGGCGATACCTAAAGC

EFI_002990_T2 TGATGCTGCTCAGGCACCAGAGCTGTTTACAACGGCTCCTGCCCTGGCGATACCTAAAGC

EFI_002990_T4 TGGGACACCCTCTAGGTTGCAGTGGAGCTCGAATCATGGTCACACTTTTGGGGGTGCTGA

EFI_002990_T3 TTTTTAGGTGTTTG---GGTTTTTG-ATCTTTTCCTTTATACCATGCTGTGGGTTTAATC

EFI_002990_T5 TTTATCAATCTCCAAGAGGTAACTGTGTCTTTCCTTTCGCATAATGAGATGAGCAGAAAA

EFI_002990_T1 ------------------------------------------------------------

EFI_002990_T6 TTTGTCAAAAGCTAG--CTTGGATGCTTCTGAAGTTGATTATTATGAGATAAATGAAGCC

EFI_002990_T2 TTTGTCAAAAGCTAG--CTTGGATGCTTCTGAAGTTGATTATTATGAGATAAATGAAGCC

EFI_002990_T4 GACACAAAAATGGGAAACATGGTGTTGCTGGTGTGTGCAATGGGGGCGGTGGTGCATCTG

EFI_002990_T3 TATGAGCCTTTTTTTTATTTGAGGT-AGTAATTA--------------------------

EFI_002990_T5 CCTGTAAATTGTAATTATTCCATGCCAACTATTGTTAATTTTCATAGGCTAATTTTCATA

EFI_002990_T1 ------------------------------------------------------------

EFI_002990_T6 TTTGC-TGTTGTAGCTCTGTCCAATCAGAAACTGCTTGGACTTAATCCAGAAAAAGTGAA

EFI_002990_T2 TTTGC-TGTTGTAGCTCTGTCCAATCAGAAACTGCTTGGACTTAATCCAGAAAAAGTGAA

EFI_002990_T4 CTCTTGTCGTAGAGCTTCTGTAGGATTTTGGGTACAAACTGATGCTGCGGTTTCTTTTCA

EFI_002990_T3 ------------------------------------------------------------

EFI_002990_T5 ATGTTGCTAGGAAGCTTGGATGCTCCATTTCCTTATTACCTGAGATTGCTACAGTTTGAA

EFI_002990_T1 ------------------------------------------------------------

EFI_002990_T6 TGTACATGGTGGAGCTGTTTCCTTGGGACACCCTCTAGGTTGCAGTGGAGCTCGAATCAT

EFI_002990_T2 TGTACATGGTGGAGCTGTTTCCTTGGGACACCCTCTAGGTTGCAGTGGAGCTCGAATCAT

EFI_002990_T4 AAAGCCAAAAGTAATCTTCAGATTTGAATGTTGTGTAATTCTACTGTTTCTATTTTTATT

EFI_002990_T3 ------------------------------------------------------------

EFI_002990_T5 TTATGACAAGAAGATTATTTTTTGTACTTCTATTCTACAGTTTGAATTTGC---------

EFI_002990_T1 ------------------------------------------------------------

EFI_002990_T6 GGTCACACTTTTGGGGGTGCTGAGACACAAAAATGGGAAACATGGTGTTGCTGGTGTGTG

EFI_002990_T2 GGTCACACTTTTGGGGGTGCTGAGACACAAAAATGGGAAACATGGTGTTGCTGGTGTGTG

EFI_002990_T4 TTATTTTTGGCTTTGGATCTCTAATAAACTACTAGTGTAACTTTGAAAGAAAATATATCT

EFI_002990_T3 ------------------------------------------------------------

EFI_002990_T5 ------------------------------------------------------------

EFI_002990_T1 ------------------------------------------------------------

EFI_002990_T6 CAATGGGGGCGGTGGTGCATCTGCTCTTGTCGTAGAGCTTCTGTAGGATTTTGGGTACAA

EFI_002990_T2 CAATGGGGGCGGTGGTGCATCTGCTCTTGTCGTAGAGCTTCTGTAGGATTTTGGGTACAA

EFI_002990_T4 GAGATCTGTAGGCTTGTTTGTGAGGAATTTATAATTTAACTAATAAATGGTGAATCAGAA

EFI_002990_T3 ------------------------------------------------------------

EFI_002990_T5 ------------------------------------------------------------

EFI_002990_T1 ------------------------------------------------------------

EFI_002990_T6 ACTGATGCTGCGGTTTCTTTTCAAAAGCCAAAAGTAATCTTCAGATTTGAATGTTGTGTA

EFI_002990_T2 ACTGATGCTGCGGTTTCTTTTCAAAAGCCAAAAGTAATCTTCAGATTTGAATGTTGTGTA

EFI_002990_T4 AAAGGCAACCCAGAAAGAAAAAAATAATTTGATTTGCCAGGGATTAATTAGCCATTCCTG

EFI_002990_T3 ------------------------------------------------------------

EFI_002990_T5 ------------------------------------------------------------

EFI_002990_T1 ------------------------------------------------------------

EFI_002990_T6 ATTCTACTGTTTCTATTTTTATTTTATTTTTGGCTTTGGATCTCTAATAAACTACTAGTG

EFI_002990_T2 ATTCTACTGTTTCTATTTTTATTTTATTTTTGGCTTTGGATCTCTAATAAACTACTAGTG

EFI_002990_T4 CCCACCCG----------------------------------------------------

EFI_002990_T3 ------------------------------------------------------------

EFI_002990_T5 ------------------------------------------------------------

EFI_002990_T1 ------------------------------------------------------------

EFI_002990_T6 TAACTTTGAAAGAAAATATATCTGAGATCTGTAGGCTTGTTTGTGAGGAATTTATAATTT

EFI_002990_T2 TAACTTTGAAAGAAAATATATCTGAGATCTGTAGGCTTGTTTGTGAGGAATTTATAATTT

EFI_002990_T4 ------------------------------------------------------------

EFI_002990_T3 ------------------------------------------------------------

EFI_002990_T5 ------------------------------------------------------------

EFI_002990_T1 ------------------------------------------------------------

EFI_002990_T6 AACTAATAAATGGTGAATCAGAAAAAGGCAACCCAGAAAGAAAAAAATAATTTGATTTGC

EFI_002990_T2 AACTAATAAATGGTGAATCAGAAAAAGGCAACCCAGAAAGAAAAAAATAATTTGATTTGC

EFI_002990_T4 ------------------------------------------------------------

EFI_002990_T3 -------------------------------

EFI_002990_T5 -------------------------------

EFI_002990_T1 -------------------------------

EFI_002990_T6 CAGGGATTAATTAGCCATTCCTGCCCACCCG

EFI_002990_T2 CAGGGATTAATTAGCCATTCCTGCCCACCCG

EFI_002990_T4 -------------------------------

**B) Example of multiple sequence alignment of an Oases ‘isoform cluster’**

| **Sequence ID** | **Blast hit ID** | **Blast hit description** | **E-value** |
| --- | --- | --- | --- |
| EFI_002990_T1 | XP_002532204 | acetyl-CoA acetyltransferase, mitochondrial, putative [*Ricinus communis*] | 5.00E-44 |
| EFI_002990_T2 | BAF98277 | acetyl-CoA C-acetyltransferase [*Hevea brasiliensis*] | 1.00E-175 |
| EFI_002990_T3 | XP_002532204 | acetyl-CoA acetyltransferase, mitochondrial, putative [*Ricinus communis*] | 1.00E-118 |
| EFI_002990_T4 | BAF98276 | acetyl-CoA C-acetyltransferase [*Hevea brasiliensis*] | 4.00E-96 |
| EFI_002990_T5 | XP_002532204 | acetyl-CoA acetyltransferase, mitochondrial, putative [*Ricinus communis*] | 1.00E-118 |
| EFI_002990_T6 | BAF98276 | acetyl-CoA C-acetyltransferase [*Hevea brasiliensis*] | 0 |
