# Supplementary material for: De novo assembly of Euphorbia fischeriana root transcriptome identifies prostratin pathway related genes
Source: BMC Genomics. 2011 Dec 13;12:600. doi: 10.1186/1471-2164-12-600 (PMC3273484; doi:10.1186/1471-2164-12-600)
Supplement: Additional file 6 — Clustering of GGPS with unrelated hypothetical proteins. A) Blast results of transcripts clustered into EFI_010585 isoform cluster using Oases. Note that only transcript 4 has similarity to GGPS and this is encoded in the reverse strand. B) Multiple sequence alignment of the reverse complemented GGPS transcript and a sequence representing the hypothetical transcripts. [file 1471-2164-12-600-S6.DOC]

**Additional file 6. Clustering of GGPS with unrelated hypothetical proteins.**

A) Blast results of transcripts clustered into EFI_010585 gene cluster using Oases. Note that only transcript 4 has similarity to GGPS and this is encoded in the reverse strand.

B) Multiple sequence alignment of the reverse complemented GGPS transcript and a sequence representing the hypothetical transcripts.

**A) Blast screening of EFI_010585 transcripts against NR database.**

| **Sequence ID** | **Blast hit ID** | **Blast hit description** | **E-value** |
| --- | --- | --- | --- |
| EFI_010585_T1 | XP_002523264 | hypothetical protein RCOM_0649410 [Ricinus communis | 3.00E-56 |
| EFI_010585_T2 | XP_002523264 | hypothetical protein RCOM_0649410 [Ricinus communis] | 1.00E-129 |
| EFI_010585_T2 | XP_002523264 | hypothetical protein RCOM_0649410 [Ricinus communis] | 1.00E-129 |
| EFI_010585_T3 | XP_002523264 | hypothetical protein RCOM_0649410 [Ricinus communis] | 2.00E-58 |
| EFI_010585_T3 | XP_002523264 | hypothetical protein RCOM_0649410 [Ricinus communis] | 2.00E-58 |
| EFI_010585_T3 | XP_002523264 | hypothetical protein RCOM_0649410 [Ricinus communis] | 2.00E-58 |
| EFI_010585_T4 | XP_002530137 | geranylgeranyl pyrophosphate synthase, putative [Ricinus communis] | 100 |
| EFI_010585_T5 | XP_002523264 | hypothetical protein RCOM_0649410 [Ricinus communis] | 2.00E-76 |
| EFI_010585_T6 | XP_002523264 | hypothetical protein RCOM_0649410 [Ricinus communis] | 9.00E-59 |

**B) Multiple sequence alignment of the GGPS transcript and a sequence representing the hypothetical transcripts.**

EFI_010585_T2 CATTTATCTTCTCCCCATTTCACTTAAACATTTCCCAAACAAACCAAGAAATTAATGGAC

EFI_010585_T4 ----------------------------------------------AGAAAATAAAGTAA

***** *** * *

EFI_010585_T2 GCTGATAGACCCCATCGCAGCAATAGTAGCTGCAGCTCCACCACCAATACCAATACCACT

EFI_010585_T4 GA-GAAGGAACCCTTAGCAG-ATTCATGGCTG-AGCTTCGCCGTCGTCACTAA---CATT

* ** ** *** * **** * * * **** **** * ** * ** ** ** *

EFI_010585_T2 AGCGAGCTCTTTATTTGCTTCACTTCTCGTCTCTCTTCTTCTTCCATGAAGATCTCTTCC

EFI_010585_T4 AATTAGCATAAACATAAACCCCTTTTTTCTCCATTTGCTAATTACCCAAAAATCTGAGTC

* *** * * ** * ** * * ** ** * ** **** *

EFI_010585_T2 AAGTCTATTCTTAGCCCCGGCCGATCCAGAGAATCTTCTCAAATCTCCCTTTCTACTTCC

EFI_010585_T4 GGTTATATGTTGTTCTTTTGCGGGTTT------CCTTCTTTGACTCTTCTTCTTCTTCTT

* *** * * ** * * ***** * *** * *

EFI_010585_T2 CTTAGCCGGAGATTGAGAACCAATGGCAGCATGAGAGGCGGTCAAGCTTCCCCTATGTTC

EFI_010585_T4 CTTCNNNNNNNNNNNNNNNNNNNNNNNNNNNNNNNNNNNNNNNNNNNNNNNNNNNNNNNN

***

EFI_010585_T2 CCTACTAACAACGGCAAGAAACGTGGCTCTTTTGAAAATCCTGAACCTTCTTCTCCTAAA

EFI_010585_T4 NNNNNNNNNNNNNNNNNNTCGCTTCCTTTGATCAGCAGCAGTAAAG--TAAAACACAGGC

* * * * * * ** * *

EFI_010585_T2 GTCACCTGCATCGGTCAAGTCAGGGTTAAGACCAAGAAACAGGGTAAGAAAATGAG-GTC

EFI_010585_T4 GGCGGCGATGTTATTCACGCGGGGGTTATCACGCATTTCGAGAACTTGCAGCCGTTTGTT

* * * * *** * ****** ** * ** * * * **

EFI_010585_T2 CAGATCTCAACGGAGATCAGGCGAAGTCAGTTTTAGAAGAGTCGAACATGCTAAAACTTG

EFI_010585_T4 TCTTTCTCAGCGAATA-CATCTTTCTCTTCCTTCCGAGAATTCCCATTT-----AATTGG

***** ** * * ** ** ** * ** * * ** * *

EFI_010585_T2 TAATCTTGAAGATGATTTCGCCCATTCTCATCTTGAAAACCAGTTTCTAAATCATTCTCA

EFI_010585_T4 AGATTCTACGCATAAGGTT-TTCAATCGCAG---AGAAACTTATATCTGGAGCTTACCTG

** * ** * * ** ** ** **** * *** * * * *

EFI_010585_T2 TTCTCACCATCAACAGCACGAGTGCTCGCCTCATAGGAATCAGAAATGGGTGCATTTGCC

EFI_010585_T4 CTTTGCATGGCTTTAAACAACATATTCATCAGACCAGC-TCCTTAATCGAGGAACAA---

* * * * * ** * * * ** *** * * *

EFI_010585_T2 TTTGACTATATGTGAAGCTTTGAAGTTCAATTGCTTTTTGCCTTGCCGCTCATCTTGTAC

EFI_010585_T4 TCTGACCCATTTTCCCTCGTTGCTGATGAACTATCACTA--CTTGCTAAT-AGGTTGCG-

* **** * * * *** * * ** * * ***** * * ***

EFI_010585_T2 GGCGACCGAGAAAGACAAGGAAGAGAAGCCGGCCAGCAATGGAAGTAGTAGCTCTTGCGG

EFI_010585_T4 GGCAATGGTGGTTGCCGAGGTCCCTAAGCTCGCCTCTGCTGCTG--AGTACTTCTTCAAG

*** * * * * * *** **** *** ** **** **** *

EFI_010585_T2 CGCGGTGTTTGCCAGGTGGCTGGTGGCGGTTCAAGAGGGAGGTGGAGATGGAGATGGAGA

EFI_010585_T4 ATGGGAG--TAGAAGGAAAGAGGTTTCGTCCTACGGTTTTGTTGCTTATGGC-ATCAGCT

** * * *** *** ** * * * ** **** **

EFI_010585_T2 TGGAAAGAGAAGAGAGATCGAGTTAGTGGTTGGTGGAGGAGAAGAAG--AAGAAGANNNN

EFI_010585_T4 TTGAATGTGGGCATACCTAGAAC-GTTGCCAAATAACATTGAAGATGCTATGGAAACAGA

* *** * * * * * ** ** * ***** * * * * *

EFI_010585_T2 NNNNNNNNNNNNNNGGAAGAGAGGAGGAGAAGCTACAGGAGACATGTTTTTGAAGAGATT

EFI_010585_T4 ATTACGAACAAGACAGCAACGTATAGCTGAAATTACAGAAATGATACATGTGGCAAGTCT

* * * ** *** ***** * ** * ** ** *

EFI_010585_T2 GATTTCAAGGAAGAGAAATATGAACATGTGCAAGAAGAAGAGGAAGCCAGGGTCAGTATT

EFI_010585_T4 --TCTC------------CATGATGATGTGTTGGACGATGCAGACACAAGGCGTGGCATT

* ** **** ***** ** ** * ** * *** * ***

EFI_010585_T2 TGTATCCCACCCAAGAATGCTTTGTTATTGATGAGATGCAGATCTGATCCTGTCAAAATG

EFI_010585_T4 GGT---TCATTAAATTTTG--TAATGGGGAATAAGATATCAGTATTAGCCGG--AGATTT

** ** ** ** * * ** **** * * * ** * * * *

EFI_010585_T2 GCTGCTCTTGCTAACAAATTCTGGGAACCACCTCTTCTCAATGATGAGGATGTGGAAGAC

EFI_010585_T4 TCTGCTTTC---AAGAGCTTGTGTGGCACTTGCTTCCTTGA-----AGAATACAGAGGTT

***** * ** * ** ** * * * ** * ** ** ** *

EFI_010585_T2 AAA-CACAAAGAACAACAAGAAAAGAGTAATAATGTTGCAGAAGAAGAGAGGCTAATTGA

EFI_010585_T4 GTCTCCCTTTTAGCGACAGTGGTAGAGCA----TCTTGTA--ACAGGCGAAACCA--TGC

* * * * *** **** * * *** * * * * ** * * **

EFI_010585_T2 GGAAGAAAAGTTGGTTTCTTGCGATGCTGTTGAAGAAGCAGCAGCTCAAGTTATTACAGA

EFI_010585_T4 AGATGACTAGTACATATGA-GCAACGTTGTAGCATGG-----AGTACTATATGCAAAAGA

** ** *** * * ** * * *** * * ** * * * * ***

EFI_010585_T2 GAAACAATGGGATTCCGTGCAGGAACAACATCAAACAGAAATTCAAGAAAATCAAGATCC

EFI_010585_T4 CATACTACAAGACT----GCATCTTTGATTTCAAACAG--CTGCAAAGCAATTGCACTTC

* ** * ** * *** * ******** * *** *** * *

EFI_010585_T2 AGAGAGCATCATTGAAGAGAATGCAATCCAAGAAAATGCAATTCAAGAAAGTGAAATCCA

EFI_010585_T4 --------TCGCTGGGCAAACTGCA------GAAGTTTCGATTTTGGCTTTTGAGTACGG

** ** * * **** *** * * *** * *** *

EFI_010585_T2 AGAAAGTGAACAAGAAAGCAATTTGACTGAGAATTCAGTGCAGTTAGAGTCACAAGAATC

EFI_010585_T4 CAAAAATCTGGGATTG-GCATTTCAGTTGATAGAT-GACGTCCTTGATTTTACAGGCACA

*** * * *** ** *** * * * ** * *** * *

EFI_010585_T2 TGGAGTAATACAAGAGCAGGAATTAGAAGACCAAGAATCCAAATCAGAAACAGAAGAAGA

EFI_010585_T4 TCAACTTCCCTTGGA---------AAGGGTTCATTATCCGACATTCGCCATGGGATTGTG

* * * ** * * ** * * * ** * * * *

EFI_010585_T2 AGAGTTACCAATACAAGAGTCAAAAGAGCATAAAGATGAAACAGAGAATAACAGCAGCCA

EFI_010585_T4 ACAGCT-CCGATATTGTTTGCTATGGAG----GAGTTTCCTCAATTACGTGCAGTTGTTG

* ** * ** *** * * *** ** * ** * *** *

EFI_010585_T2 AGTAGAGAATGAAGAAGAAGAAGAAGAAGAAGAGACATTGACCCATGGAAGATCCGAACC

EFI_010585_T4 AGGAGGGCTTTGACAAACCTGAGAATGTT----GATACTG--CCTTGGAGTACCT-----

** ** * * * ** **** ** * ** ** **** * *

EFI_010585_T2 CGAAGAAAACCTAAAAACTCAACAACGTCAAGCGGATATCCAGTCAAAGGAGAGAGAGAG

EFI_010585_T4 TGGAAAGAGCCGAGGAATACAGAGG----ACGCGGG-----AGCTAGCTGCGA-----AG

* * * * ** * ** ** * **** ** * * ** **

EFI_010585_T2 TCAGCCATTATTACCGGATTGCTTACTGTTAATGATGTGCGAGCCGAAGCTATCAATGGA

EFI_010585_T4 CATGCTAGTCTCGCCG------CTGAAGCCATTGATTCACTGCCTGAATCCGACGACGAA

** * * * *** * * * **** * * *** * * * * *

EFI_010585_T2 GGTATCCAAGGAGACATGGGTTTGCAGTA-CGGATTTCATCAGATGGTTGCCGGAGCCTT

EFI_010585_T4 GATGT--AAGAAGGTCGAGGCGGGCACTAGTAGACCTTAC--------TCACAGAGTAAT

* * * *** ** ** *** ** ** * * * * *** *

EFI_010585_T2 CAAGGCCGGTCAAGAAAAAGAGCGGTGGCGGTGGAGATGAGCCCAAGAAAAGGAGCAGCA

EFI_010585_T4 CA--------CAAGAAATAAGTGACAGAAGGATTTTCCAATTCTCAACAGCTCTTTATTA

** ******* * * ** * * * * * *

EFI_010585_T2 TCGACATTCA--TCCACCGTCGGTGCACAGTAAGTTGCAGCAGCCACCAAGGTCTTCTTG

EFI_010585_T4 TAGATTTTTGGTTTCAATTTTTATTTCTTGTAGACAACACTCTTCTTTATAGTTTCATTG

* ** ** * ** * * *** ** * * ** * ***

EFI_010585_T2 TTCATATCCGGCCAAGCCGCCGCCTCGTG-CAGCCGGCGCTGA--ATCTATGTCGACAGC

EFI_010585_T4 ATTTGATTACGTTAA-TTGTTGTTTAATGGTAACTTTTGCGGACGATTCATTTTGTAAAT

* ** * ** * * * ** * * ** ** ** ** * * *

EFI_010585_T2 AATAGAGCAGAAGCTGGTTGGAAGC-AAATCTTGTGAGCCGTTCATGCTTAAGCGCTGCA

EFI_010585_T4 TATAGGTCTCGTGGAGAAGGGCAACGAAATAATAGTCACAGGTTTTGCTCGTATGTTGTG

**** * * * ** * * **** * * * * **** * **

EFI_010585_T2 AGTCGGAGNNNNNNNNNNNNNNNNNNNNNNNNNNNNNNNNNNNNNNNNNNNNNNNNNNNN

EFI_010585_T4 TAACGAAATCTGTTCCGGCAAAACGGGTATCGAAGTTCCTGAACTTCTTTTAACTGTTTA

** *

EFI_010585_T2 NNNNNNNGAGAATTGTTTTTGGAAGAACAGGAAGCTGGAGCCGCATCGTCCGGCTACGTT

EFI_010585_T4 TGCG--------------------------------------------------------

EFI_010585_T2 TGGAGTCGGCGCGGCTGGGGTTGGATGTTAGAAATCACAAAATAATGTGTGGTAAGTTGT

EFI_010585_T4 ------------------------------------------------------------

EFI_010585_T2 AGTGTGGTAAACCGTAAAAGTGTAATGTAGTTTGACAGCTGTGATATCCCTGTAAATTTA

EFI_010585_T4 ------------------------------------------------------------

EFI_010585_T2 TTTTCCATGATATTTTAGCAAATTCATTTTTTTTCCCCTTCAGTGTAAATTTTTTTGGTG

EFI_010585_T4 ------------------------------------------------------------

EFI_010585_T2 AATTTTGTC

EFI_010585_T4 ---------
