# Supplementary material for: De novo assembly of Euphorbia fischeriana root transcriptome identifies prostratin pathway related genes
Source: BMC Genomics. 2011 Dec 13;12:600. doi: 10.1186/1471-2164-12-600 (PMC3273484; doi:10.1186/1471-2164-12-600)
Supplement: Additional file 7 — Predicted tRNA genes in the E. fischeriana root transcriptome. To identify tRNA genes the reference assembled root transcriptome was screened using tRNAscan-SE as previously described [17]. Additionally, we identified another four tRNA genes highlighted with asterisks in a transcriptome assembly conducted using a k-mer size of 17 and a length threshold of > = 100 bp. Their fasta sequences are appended at the bottom. [file 1471-2164-12-600-S7.DOC]

**Additional file 7: Predicted tRNA genes in the *E. fischeriana* root transcriptome.** To identify **t**RNA genes the reference root transcriptome was screened using tRNAscan-SE as previously described [17]. Additionally, we identified another four tRNA genes highlighted with asterisks in a transcriptome assembly conducted using a k-mer size of 17 and a length threshold of >= 100bp. Their fasta sequences are appended at the bottom.

| **Transcript ID** | **tRNA #** | **tRNA Begin** | **Bounds End** | **tRNA type** | **Anti codon** | **Coverage score** |
| --- | --- | --- | --- | --- | --- | --- |
| EFI_002280 | 1 | 163 | 235 | Glu | TTC | 58.03 |
| EFI_002280 | 2 | 306 | 389 | Tyr | GTA | 55.88 |
| EFI_002280 | 3 | 733 | 806 | Asp | GTC | 69.76 |
| EFI_003197 | 1 | 680 | 607 | Pro | TGG | 73.89 |
| EFI_003197 | 2 | 411 | 338 | Trp | CCA | 76.17 |
| EFI_014332 | 1 | 117 | 188 | Gln | TTG | 62.47 |
| EFI_006582 | 1 | 136 | 65 | Asn | GTT | 72.21 |
| EFI_008905 | 1 | 396 | 325 | Pseudo | ATG | 24.31 |
| EFI_017567 | 1 | 192 | 121 | Arg | TCT | 65.86 |
| EFI_009712 | 1 | 219 | 148 | Pseudo | GCT | 36.33 |
| EFI_018181* | 1 | 90 | 17 | Met | CAT | 69.14 |
| EFI_018182* | 1 | 82 | 169 | Ser | GCT | 64.41 |
| EFI_018183* | 1 | 131 | 203 | Phe | GAA | 73.29 |
| EFI_018184* | 1 | 297 | 209 | Ser | GGA | 23.24 |

Fasta sequences of the 4 additional tRNAs are shown below:

>EFI_018181

TTTTACAAAAAAAATATTGCGGAGACGGGATTTGAACCCATGACTTCAAGGTTATGAGCCTTGCGAGCTACCAAACTGCTCTACCCCGCGTGATGACGCGAAGAACTCGGAACTAATCTAGTGGACAAACAAGGCTTGAATGTGCCCCCCTACCATATCTGTACAAATAGAATAG

>EFI_018182

CTTTTTTTGTCAAGCCTTACTTGTTGTATAATGTAACTACTTGCTCTGTAATGTAACATAATAATTATCCTTTTTCAATCGGGAGAGATGGCTGAGTGGACTAAAGCGTCGGATTGCTAATCCGTTGTACGAATTATCCGTACCGAGGGTTCGAATCCCTCTCTTTCCGGTTCCAGTGATGACTTGAATTTTTTTTATCTTTTCTTTCAAA

>EFI_018183

AAAGATAAGATACAAAACATTTCAGGTCTGGGTAAGACTTTAGAATATTTTTTCGTCTTTTTTAAATTGACATAGGCCCAAGTTTTTTACTAAAATTTAGTAAAACGAGGAAGACGACGCGGCGAAAATGGTCGGGATAGCTCAGTTGGTAGAGCAGAGGACTGAAAATCCTCGTGTCACCAGTTCAAATCTGGTTCCTGGCACATGATTTATTTGTGTATTAAGTATCCATTCTACAATTTAGTGAAATTTCGATCTGATATAGGTCAACATAGATATTCAGTAATGGTATGGATG

>EFI_018184

TTCCTCTACATTTTTTCCAGTATTTTCTTTTCTGTACCGCAGAAATTAGGAATAGAGAATCTCTATCAAGGATCAAGGAANNNNNNNNNNNNNNNNNNNNNNNNNNNNNNNNNNNNNNNNNNNNNNNNNNNNNNNNNNNNNNNNNNNNNNNNNNNNNNNNNNNNNNNNNNNNNNNNNNNNNNNNNNNNNNNNNNNNNNNNNNNNNNNNNNNNNNNNNNNNNNNATTCGAACCCTCGGTAAACAAAAGCCTACATAGCAGTTCCAATGCTACGCCTTGAACCACTCGGCCATCTCTCCTACATAATGATTATGTCCCAGAAACTGAGTGAATCGCGAATCATTCGTATTAGATTGTTGCGTAGATATGGTACGTACAATCAATTCTAACTAGAA
