# Supplementary material for: De novo assembly of Euphorbia fischeriana root transcriptome identifies prostratin pathway related genes
Source: BMC Genomics. 2011 Dec 13;12:600. doi: 10.1186/1471-2164-12-600 (PMC3273484; doi:10.1186/1471-2164-12-600)
Supplement: Additional file 8 — Predicted rRNA genes the E. fischeriana root transcriptome. To find rRNAs the reference root transcriptome was screened using RNAmmer as previously described [18]. [file 1471-2164-12-600-S8.DOC]

**Additional file 8: Predicted rRNA genes the *E. fischeriana* root transcriptome. To find rRNAs the reference root transcriptome was screened using RNAmmer as previously described [18].**

| **Transcript ID** | **rRNA Start** | **rRNA End** | **Direction (Strand)** | **rRNA molecule** | **Score** |
| --- | --- | --- | --- | --- | --- |
| EFI_006729 | 67 | 181 | - | 8s | 22.3 |
| EFI_006898 | 172 | 286 | - | 8s | 50.2 |
| EFI_000286 | 1 | 1536 | + | 28s | 158.4 |
| EFI_000775 | 73 | 2870 | + | 28s | 84.1 |
| EFI_000294 | 1 | 1684 | - | 18s | 1103.0 |
| EFI_010542 | 1 | 1898 | + | 18s | 50.0 |
